# Assessment of the Quality of Polluted Areas in Northwest Romania Based on the Content of Elements in Different Organs of Grapevine (*Vitis vinifera* L.)

**DOI:** 10.3390/molecules25030750

**Published:** 2020-02-09

**Authors:** Florin Dumitru Bora, Claudiu Ioan Bunea, Romeo Chira, Andrea Bunea

**Affiliations:** 1 Research Station for Viticulture and Enology Târgu Bujor, Department of Physico-Chemistry and Biochemistry, 805200 Târgu Bujor, Romania; boraflorindumitru@gmail.com; 2 University of Agricultural Sciences and Veterinary Medicine, Department of Horticulture and Landscaping, 3-5 Mănăştur Street, 400372 Cluj-Napoca, Romania; claus_bunea@yahoo.com; 3 University of Medicine and Pharmacy “Iuliu Hatieganu”, 3–5 Clinicilor Street, 400006 Cluj-Napoca, Romania; romeochira@yahoo.com; 4 University of Agricultural Sciences and Veterinary Medicine, Department of Chemistry, 3-5 Mănăştur Street, 400372 Cluj-Napoca, Romania

**Keywords:** heavy metals, grapevine, bioaccumulation, biomonitoring

## Abstract

The purpose of this study was to evaluate the environmental quality of polluted areas near the Baia Mare Mining and Smelting Complex for future improvements the quality of the environment in polluted areas, such as the city of Baia Mare and its surroundings. Samples of soil and organs of grapevine (*Vitis vinifera* L.) were collected from Baia Mare, Baia Sprie and surrounding areas (Simleul Silvaniei) and their content of Cu, Zn, Pb, Cd, Ni, Co, As, Cr, Hg were analyzed. Most soil and plant samples showed higher metal concentrations in Baia Mare and Baia Sprie areas compared to Simleul Silvaniei, exceeding the normal values. The results obtained from the translocation factors, mobility ratio, as well as from Pearson correlation study confirmed that very useful information is recorded in plant organs: root, canes, leaves and fruit. Results also indicated that *Vitis vinifera* L. has some highly effective strategies to tolerate heavy metal-induced stress, may also be useful as a vegetation protection barrier from considerable atmospheric pollution. At the same time, berries are safe for consumption to a large degree, which is a great advantage of this species.

## 1. Introduction

Pollution is a worldwide problem caused by anthropogenic activities such as mining, petrochemical refining, and smelting, with negative impacts on human health. In Romania, 18% of population was exposed to heavy environmental pollution whereby serious health risks are likely. A total of 14 environmental pollution “hot spots” have been identified in Romania: Copșa Mică, Baia Mare, Ploiești-Brazi, Zlatna, Onești, Bacău, Suceava, Petești, Târgu Mures, Turnu Măgurele, Talcea, Isalnita, Brașov, and Govora; 5.3% of the population lives in these heavily polluted areas, mostly in the critical rural/urban interfaces [1,2,3].

Growing in extremely polluted areas, some plant species can be seriously damaged, whereas others can survive without any visible changes [4]. Uptake of trace metals by plants can happen from the soil through the roots and subsequent transport to the leaves or directly from the air. Specific mechanisms allow plant tissues to accumulate high quantities of trace metals, playing, thereby, a vital role in the natural recovery of industrial damage [5]. In this respect, trees are especially useful because contaminants can accumulate in their large biomass and they can grow in soil with poor fertility and structure [6]. Terrestrial higher plants are specific living-system structures with unique ecobiological characteristics. They interact actively with three spheres: soil, water and air, at the same time, requiring only modest nutrient input. Along with nutrients, plant roots can absorb a range of anthropogenic toxic materials. Heavy metals are just a class of such pollutants and several of them are well known as nonessential and extremely toxic for plants: cadmium (Cd), lead (Pb), mercury (Hg) and arsenic (As). Even essential micronutrients such as copper (Cu), zinc (Zn), and nickel (Ni) may become toxic for plants when absorbed above certain threshold values [7].

Plants have developed effective detoxification mechanisms to manage heavy metal content [8]. Some species may concentrate heavy metals in root cell walls and/or vacuoles, thus minimizing their phytotoxicity [9] and also preventing the spread of these contaminants in soil [10]. Phytoremediation is an excellent opportunity for cleaning up the pollute environment in an economic and ecological friendly manner. It uses green plants to detoxify the polluted environment, and it may be applied in a variety of ways [10]. On the other hand, plants can be used as indicators of the pollution level of the environment. Heavy metals in plant organs, especially in roots and leaves, represent a very specific evidence of spatial and temporal history of polluted area [11]. Researchers agree that the root and leaf analyses are essential in the evaluation process of the environmental quality of ecosystems or to study the effects of heavy metals on the chemical composition of plants.

Grapevine is an important crop worldwide, while the wine sector is of major importance for the economy of many countries [12]. The soil chemistry in vineyards influences wine and grape quality, vine-soil relationship being a key part of the concept of terroir [13,14]. The town of Baia Mare has been an important nonferrous metallurgical center where heavy metals like Pb and Cu have been extracted and processed for centuries. Metallurgical plants ”Romplumb”, located in the Ferneziu district, and ”Cuprom”, located in the eastern part of the city, had polluted the soil in Baia Mare area with Pb, Cd, Cu, Zn, and As [15,16,17].

In this study, concentration of Cu, Zn, Pb, Cd, Ni, Co, As, Cr and Hg in vineyard soil, several parts of grapevine (*Vitis vinifera* L.), as well as in must and wine from Baia Mare, Baia Sprie, and Simleul Silvaniei areas were analyzed.

## 2. Results and Discussion

### 2.1. Metal Concentration in Soil Samples

Elemental concentration varied among soil samples but were considerably higher than concentrations allowed by the Romanian Regulation of allowable quantities of hazardous and harmful substance in soil (Order of the Ministry of Waters, Forests and Environmental Protection No. 756/3 November 1997), as well as by the Council Directive 86/278/EEC for Protection of the Environment (European Communities Council 1986) (Table 1). Physical properties of soil samples are provided in Supplementary Table S1.

Regardless of sampling depth, the highest concentrations of Cu were recorded in Baia Sprie area, followed by the Baia Mare area and Simleul Silvaniei area (Table 1). In all cases, the concentrations significantly exceeded the normal values set by the corresponding legislation (20 mg/kg). These high concentrations can be attributed to the pollution factor (in Baia Mare and Baia Sprie areas) or the extensive usage of Cu-based plant protection products (in the Simleul Silvaniei area). Detected values were higher than those reported previously from this area (640.6 mg/kg) [17], (599.75 mg/kg) [18], (314.00 mg/kg) [16] or other wine-producing areas in Southeast Romania [19], but were within the range established for Copșa Mică (77–7675 mg/kg) [3]. 

The results obtained by Damian et al. (44–5823 mg/kg) are comparable to those obtained in this research. The Cu values obtained for Simleul Silvaniei are conformable with those recorded by Alagić et al. (293.00 mg/kg) [21] and Bora et al. (479.64 mg/kg) [23].

In the Baia Sprie and Baia Mare areas, the concentrations of Zn tended to increase with the sampling depth, with the highest concentrations being detected in samples collected at 60–80 cm (3483.25 ± 94.11 mg/kg and 2734.93 ± 147.45 mg/kg, respectively). All values greatly exceeded the normal levels of Zn allowed by the law (100 mg/kg). In contrast, in the Simleul Silvaniei area, the highest concentration was recorded in the surface soil profile (76.86 ± 7.71 mg/kg (0–20 m)), and it tended to decrease with the increasing soil depth. The Zn values obtained are higher than data published in previous reports from Baia Mare or other Romanian regions [16,26,27].

Concentrations of Pb and Cd varied within a wide range (Table 1). The highest concentrations were recorded in the Baia Mare and Baia Sprie areas, significantly higher than those detected in Simleul Silvaniei or allowed by the applicable legislation (20 mg/kg for Pb, 1 mg/kg for Cd). The extremely high values of Pb and Cd indicate severe heavy metal pollution in these two areas. Similar [18] or lower [16,27] values were also reported from these regions. The average content of Ni and Co in soil samples exceeded the normal concentration in the Baia Mare area (25.29 ± 2.07 vs 20 mg/kg for Ni and 22.57 ± 1.65 vs 15 mg/kg for Co), but were below the limit in the other two areas (Table 1). For comparison, Mihali et al. recorded similar values (13.1 mg/kg [Ni] and 24.8 mg/kg [Co]) in the Baia Mare area [19], while another study conducted in unpolluted regions from Dobrogea and Muntenia reported Ni concentrations between 0.97–11.29 mg/kg and Co concentrations between 0.49–4.36 mg/kg [26].

The concentrations of As, Cr, and Hg indicated no pollution of the soil samples with these heavy metals; values were below the normal levels. The highest values were obtained in Baia Sprie (4.46 ± 1.56 mg/kg As; 2.31 ± 0.78 mg/kg Cr; 0.064 ± 0.016 mg/kg Hg) followed by Baia Mare (3.46 ± 0.63 mg/kg As; 2.51 ± 0.51 mg/kg Cr; 0.052 ± 0.021 mg/kg Hg) area. A recent study conducted in Vaslui county reported higher content of As (10.14 mg/kg) and Cr (62.05 mg/kg) compared to our results [28].

### 2.2. Metal Concentration in Plant Material Samples

#### 2.2.1. Metal Concentration in Roots

Roots are in direct contact with the soil solution and the concentration of heavy metals in roots is generally used as indicative of soil metal bioavailability [29]. Varieties cultivated in Simleul Silvaniei showed the lowest concentrations of Cu and Zn, compared to the varieties from Baia Mare and Baia Sprie. Italian Riesling from Baia Mare and Baia Sprie (779.15 ± 4.66 mg/kg and 670.51 ± 6.61 mg/kg, respectively) and Feteasca alba from Baia Sprie (669.15 ± 21.27 mg/kg) contained the highest concentration of Cu while the varieties cultivated in Baia Mare area recorded the highest Zn concentration (Table 2). Studies have shown that high concentration of Cu can affect the growth of the roots [9,30]. The highest concentrations of Pb was registered in Feteasca regala from Baia Mare (60.81 ± 5.95 mg/kg), in Italian Riesling from Baia Sprie (92.26 ± 1.11 mg/kg), significantly higher as compared to the same varieties grown in the Simleul Silvaniei area (0.83 ± 0.60 mg/kg (Feteasca regala) and 0.43 ± 0.17 mg/kg (Italian Riesling)). According to Vamerali et al., Pb has no important role in functions of plants [7]. Roots of Feteasca regala from Baia Mare and Feteasca alba from Baia Sprie had the highest concentration of Cd (7.09 ± 0.83 mg/kg and 3.07 ± 0.12 mg/kg, respectively) and Co (32.24 ± 1.23 mg/kg and 10.95 ± 1.26 mg/kg) (Table 2). Cd and Co concentrations in these areas were significantly higher than those obtained in varieties grown from Simleul Silvaniei. In Baia Sprie and Simleul Silvaniei, concentrations of Ni and As were similar amongst varieties, while in Baia Mare area, Italian Riesling variety had higher concentration of As compared to Feteasca alba and Feteasca regala (Table 2). Interestingly, roots from Simleul Silvaniei showed higher content of Ni compared to varieties from Baia Sprie. Concentration of Cr was similar for all three varieties in Baia Mare and Baia Sprie, except for Feteasca regala from Baia Mare which recorded a significantly higher concentration.

The varieties grown in Simleul Silvaniei have a lower concentration compared to varieties grown in Baia Mare and Baia Sprie. Hg was detected in low concentrations in all three areas. The observed concentrations of Cu, Zn, Ni, and As exceed the toxic threshold in plant tissues [7,31]. Overall, data suggests that high concentrations of heavy metals in soil result an increased metal content in the roots as well. Compared to our findings, grapevines grown in polluted areas from East Serbia have shown similar concentrations of heavy metals in roots [21].

#### 2.2.2. Metal Concentration in Canes

Cu has the highest concentrations in all varieties cultivated in Baia Sprie (147.92 ± 2.46 mg/kg (Italian Riesling); 143.72 ± 2.46 mg/kg (Feteasca Regala) 124.56 ± 9.02 mg/kg (Feteasca Alba), followed by the Baia Mare area (77.31 ± 3.76 mg/kg (Feteasca Regala); 72.93 ± 2.15 mg/kg (Italian Riesling); 72.03 ± 2.75 (Feteasca Alba). This can be explained with the heavy metal pollution phenomenon. Though Cu is involved in many vital processes in plants such as photosynthesis, flowering, seed production, and plant growth, its excessive concentrations may cause a significant modification of biochemical processes, leading to the reduction of shoot growth [7,32]. Results obtained in Baia Mare are comparable with those reported from Turulung, NW Romania (63.67 ± 2.67 mg/kg) [32] and much lower than those obtained from polluted regions from East Serbia (170.90 ± 0.80 mg/kg Cu [Flotacijsko Jalovište]; 175.00 ± 2.00 mg/kg [Bolničko naselje]; 160.00 ± 0.90 mg/kg [Slatinsko naselje]) [21]. Analyzing the concentration of Zn in the canes, varieties cultivated in Baia Sprie had the highest concentrations. Cane samples from the Baia Mare area also displayed high concentrations of Zn, exceeding the toxicity threshold in plant tissues [7,31]. The lower concentrations detected in the Simleul Silvaniei area were consistent with the literature values reported for other areas [21,23]. Regarding the concentration of Pb, Cd, Ni, and Co in the string and canes, values recorded in the Baia Sprie and Baia Mare areas are significantly higher than those recorded for the same heavy metals in the Simleul Silvaniei area (Table 2) or reported from other regions [21,23]. In all regions and varieties studied, concentrations of As, Cr and Hg were similar and below the toxicity threshold in plant tissues.

#### 2.2.3. Metal Concentration in Leaves

Agricultural crops are especially sensitive to Cu concentration. As a first signal of excessive supply of Cu, symptoms of chlorosis may occur [32]. In this study, significantly higher concentrations of Cu were detected in Baia Sprie as compared to Baia Mare. No significant differences in Cu concentrations were found between varieties cultivated in the same area, except for Simleul Silvaniei, where leaves of Feteasca regala had higher Cu content as the other two varieties tested. Similarly, Zn concentrations were highest in Baia Sprie. Feteasca alba leaves from Baia Sprie and Italian Riesling leaves from Baia Mare had significantly higher content of Zn than leaves of other varieties collected from the same area. For both Cu and Zn, concentrations were above the phytotoxic threshold. Concentrations of Pb, Cd, and Ni in leaves were similar across varieties cultivated in the same area. While Cd and Ni did not exceed the phytotoxic concentrations established for plant tissues, the concentrations of Pb in leaves collected from Baia Sprie and Baia Mare areas were greatly above the pre-defined phytotoxic concentration, which can be attributed to the pollution factor in these areas. Concentrations of Co were similar in Baia Mare and Baia Sprie areas ranging between 4.84 ± 0.88 mg/kg and 6.89 ± 1.01 mg/kg (Table 2). The levels in leaves were slightly above the normal range [25], but still below the phytotoxic concentration.

#### 2.2.4. Metal Concentration in Grapes

According to Vamerali et al., Cu is a constituent of enzymes involved in photosynthesis, in reproductive phase, and in determining the yield and quality in crops. Zn is a constituent of cell membranes and it is involved in DNA transcription, activation of enzymes, and evaluation of the yield and quality of crops [7]. Varieties cultivated in Baia Mare and Baia Sprie areas recorded comparable concentrations of Cu (8.49 ± 0.64–12.30 ± 2.39 mg/kg and 12.31 ± 1.82–14.51 ± 1.25 mg/kg, respectively) and Zn (8.52 ± 1.25–10.13 ± 1.33 mg/kg and 6.78 ± 2.14–8.91 ± 1.50 mg/kg), but values were comparable and within the normal range accepted in plant tissues (Table 2). While these concentrations can be attributed to the heavy metal pollution phenomenon in the two areas, Cu and Zn content of varieties cultivated in Simleul Silvaniei area (2.31 ± 0.77–3.38 ± 1.76 mg/kg and 1.11 ± 0.62–1.25 ± 0.53 mg/kg, respectively) can be ascribed to plant protection products or vine nutrition process. Values of the Cu and Zn concentration are higher than those reported in Brazil (79.87 ± 0.05 µg/100g grape berries - Cabernet Sauvignon and 31.56 ± 0.04 µg/100g grape berries - Merlot for Cu; 42.47 ± 0.17 µg/100g and 52.24 ± 0.74µg/100g for Zn) [33].

Although Pb occurs naturally in all plants, it has not been shown to play any essential role in their metabolism and its concentration at the level of 2–6 µg/g should be sufficient [25]. Pb has recently received much attention as a major metallic pollutant of the environment and as an element toxic to plants. Feteasca alba variety cultivated in Baia Sprie showed the highest Pb content (8.91 ± 1.50 mg/kg), other varieties from Baia Sprie and Baia Mare areas having similar Pb concentration (between 4.60 ± 0.64 and 6.80 ± 2.47 mg/kg). The varieties grown in Simleul Silvaniei recorded significantly lower concentration of Pb in grapes (Table 2). Overall, concentrations of Cd and Ni were detected in similar ranges in all three areas, though values tended to be higher in Baia Sprie and Simleul Silvaniei regions compared to Baia Mare. Cd is considered a non-essential element for metabolic processes; it is effectively absorbed by root and leaf systems and is also accumulated in soil organisms. There are evidences that an appreciable fraction of Cd is taken up passively by roots, but Cd is also absorbed metabolically [25]. There is no evidence of an essential role of Ni in plant metabolism, although several investigators suggested that Ni might be essential for plants. The essentiality of Ni for some biosynthesis of a number of bacteria has been proven. Also, its role in the nodulation of legumes and effects on the nitrification and mineralization of some OM was described [25]. Concentration of Co was higher in varieties from Baia Sprie area (3.26 ± 0.69–4.60 ± 2.54 mg/kg) as compared to Baia Mare (0.91 ± 0.06–1.26 ± 0.65 mg/kg) and Simleul Silvaniei (1.44 ± 0.29–1.93 ± 0.09 mg/kg). Co is cofactor of biosynthetic enzymatic activities essential for *Rhizobium*. Its content in plants is highly controlled by both soil factors and the ability of plants to absorb this metal [34]. In higher plants, absorption of Co by roots involves active transport [25]. Varieties from Baia Sprie had the highest As concentrations (0.83 ± 0.17–0.90 ± 0.07 mg/kg), followed by varieties from Baia Mare (0.29 ± 0.22–0.60 ± 0.30 mg/kg). No significant differences in Cr and Hg content were observed in all grape samples. The biochemistry of Hg is associated mainly with biological transformation of its compounds. However, it is not clear yet which processes are the most important in its cycling in the environment. In general, Hg content of plants is high when the Hg content of soils is also high, but this relation does not always hold. The results obtained are much higher than those reported in other studies [21,23,25,31].

### 2.3. Metal Concentration in Must and Wine

#### 2.3.1. Metal Concentration in must

Concentrations of Cu and Zn in must samples from Baia Sprie and Baia Mare areas exceeded the maximum permissible limit (M.P.L.) (10 mg/L), indicating a serious Cu and Zn pollution of the corresponding areas. Concentrations in the varieties cultivated in Simleul Silvaniei were below this threshold (Table 3). In grapevine nutrition, small quantities of Zn are taken from the soil (Zn is a trace mineral), so it is naturally present in must and wine. During alcoholic fermentation, part of the Zn precipitates due to the reducing environment and is accumulated in yeast.

Concentrations of Pb in must were significantly higher in Baia Sprie area than in the other two areas. All varieties cultivated in Baia Sprie and grapes of Feteasca regala from Baia Mare slightly exceeded the M.P.L. (0.5 mg/L). Concentrations are higher than those reported for Brazilian grapes juice (0.07 ± 0.00 µg/100 mL grape juice - Cabernet Sauvignon; 0.11 ± 0.00 µg/100 mL grape juice - Merlot) [33], but lower for grapes juice originated from polluted and nonpolluted regions from Serbia (1.81 ± 0.15 mg/kg) [35]. Grapevine can accumulate small amounts of Pb (27–125 mg/kg), with an average of 58.2 mg/kg in grapes [36].

The highest concentrations of Cd in must were recorded in varieties cultivated in Baia Sprie, significantly higher than in Baia Mare. Grapes samples from Simleul Silvaniei had Cd concentrations below the limit of detection. Cd is a natural component of must as it originates from the grapes. During fermentation, up to 90% of Cd accumulates in yeast, thus wine contains 0001–0002 mg/L [36]. Interestingly, must of Feteasca alba variety cultivated in Baia Mare had remarkably higher concentration of Ni compared to other varieties or the same variety from other areas (Table 3). Our values are higher than those obtained for Brazilian grapes (0.40 ± 0.01 µg/100 mL grapes juice - Cabernet Sauvignon; 0.69 ± 0.00 µg/100 g mL grapes juice - Merlot) and lower than concentrations reported for grape berries juice from Serbia (2.16 ± 0.78 mg/kg and 1.77 ± 0.14 mg/kg, respectively) [35]. The level of Co, in must, is under the detection limit in all analyzed samples. As is usually present in must as a consequence of herbicides and insecticides used for grape production, processing factors, and must storage conditions [37]. Feteasca regala and Italian Riesling varieties from Baia Mare and Baia Sprie had significantly higher concentration of As in must samples (48.30 ± 1.27 µg/L (Feteasca regala); 46.35 ± 2.60 µg/L (Italian Riesling) from Baia Mare and 50.34 ± 2.75 µg/L(Feteasca regala); 49.87 ± 2.36 µg/L (Italian Riesling) from Baia Sprie) than Feteasca alba variety from the same areas (33.06 ± 1.58 µg/L and 35.68 ± 3.29 µg/L, respectively). Concentrations of As are below the M.P.L. in all tested must samples. Highest concentrations of Cr were recorded in must samples from Simleul Silvaniei for all three varieties, while Hg was detected in comparable amounts.

#### 2.3.2. Metal Concentration in Wine

Concentrations of Cu and Zn exceeded the M.P.L. under applicable law (1 mg/L for Cu and 5 mg/L for Zn) for varieties cultivated in Baia Sprie and Baia Mare and were below the M.P.L. in varieties from Simleul Silvaniei (Table 3). These concentrations are higher than those obtained in wine samples from different wine-producing areas of Romania: 403.92 µg/L (Cu) and 1183.32 µg/L (Zn) in Cabernet Sauvignon from Muntenia [38]; 886.31 µg/L (Cu) and 524.65 µg/L (Zn) from Muntenia, 289.52 µg/L (Cu) and 488.20 µg/L (Zn) from Dobrogea, and 642.60 µg/L (Cu) and 426.40 µg/L (Zn) from Moldova [26]. M.P.L. for Pb concentration in wine (0.15 mg/L) was exceeded in varieties from Baia Sprie and Baia Mare, the highest value being detected in Feteasca regala variety (0.38 ± 0.16 mg/L and 0.27 ± 0.02 mg/L, respectively). In other wine-producing regions, concentration of Pb was reported at 27.36 µg/L (Feteasca Neagra, Dealu-Mare) [39], 44.68 µg/L (Muntenia), 31.93 µg/L (Dobrogea), and 49.59 µg/L (Moldova) [26]. Concentrations of Cd in wine samples from Baia Sprie and Baia Mare were recorded within 0.02–0.06 mg/L, slightly above the M.P.L (0.01 mg/L); no statistically significant differences were observed between these values. In Simleul Silvaniei area, Cd concentrations were below the detection limit. Compared to our values, much lower Cd concentrations were reported for several red wine samples from Banat, Muntenia, Oltenia, and Dobrogea regions [39]. Concentration of Ni was statistically comparable in all three areas, values varying slightly between 0.02 mg/L (Simleul Silvaniei) and 0.08 mg/L (Baia Sprie). In comparison, in other Romanian wine-producing regions, similar values were reported in white wine samples but higher concentrations for red wines [26,39]. Co levels in wine samples were below the detection limit of the analytical method. Concentrations of As varied significantly amongst areas, for all three varieties, following the trend Baia Mare >Baia Sprie >Simleul Silvaniei, however, all values were below the M.P.L. imposed by law. In case of Cr, the trend was as follows: Baia Mare >Simleul Silvaniei >Baia Sprie. Concentrations of Hg were below the detection limit, except for Feteasca alba from Baia Mare (0.11 ± 0.02 µg/L).

### 2.4. Pearson’s Correlations Between the Content of the Investigated Elements From Soil, Plant Material, Must, and Wine

The results of Pearson’s correlation analysis revealed that there is a good negative correlation between metals contents in all plant parts and the distance from the “Romplumb” and “Cuprom” smelters, except for Cr and Ni in cane, Cr, Pb, and Ni in leave, Pb, As, Ni, and Co in grape, and Cd in must and wine (Table 4). Ni content in soil correlates positively with the distance. These results demonstrate that pollution resulted from metallurgical activities affect the heavy metal content of plant parts. Content of Cu, Zn, Cd, As, Pb and Hg in all plant parts decreased as the distance from the main pollution source increased, except for Ni content. Apparently, the Co smelter is not necessarily a dominant source of pollution for Pb, Co, Cr and As. These elements can be easily assimilated from soil naturally enriched with heavy metals and could come from combustion of fossil fuels in residential areas, heavy traffic, or some agricultural practices in rural zone [21,25].

Significant positive correlation between metal level in plant and soil was detected in nearly all cases, while Co in grape, Pb in must, and Cr in grape and must showed significant negative correlations (Table 4). Although all elements in all samples, except for Cd, Ni, Co in grape and Cr in must, correlated positively with the metal content in roots, only the correlation of grape and root can be of interest as these organs reflect a real bioaccumulation [21].

Overall, the Pearson’s correlation matrix for individual elements in soil, plant material, must, and wine showed a good positive correlation between contents of individual elements (Supplementary Table S2). Ni content in soil and Cd content in grape had negative correlation with other elements. Similar results regarding Ni behavior have been reported from Serbia [21]. The low correlation coefficients observed for Ni in soil and plant parts (except leaves) might indicate that this element comes from different sources: Ni concentration in soil is impacted predominantly by geology, and the soil is mainly the source of Ni in plants parts. Leaves of grapevine from Baia Mare and Baia Sprie have captured Ni from atmospheres as well, originating from metallurgical activities. It is a known fact that above-ground plant parts assimilate elements from both soil and atmosphere, however, leaves are likely to be the most sensitive to air pollution.

### 2.5. Translocation Factor (TF) and Mobility of the Element Content in the Soil-Grapevine-Wine System

TF of the metals from the soil to the aerial parts of the plant represent an essential indicator of heavy metal mobility and translocation to the edible parts of the plant. Mobility ratio (MR) in *Vitis vinifera* L. was used to determine the ratio between the metal concentration in plant parts (canes, leaves and grapes) and the concentration levels of the acid-soluble metal faction in top soil. MR >1 indicates that the plants enrich these elements (accumulator), a ratio at around 1 indicates a rather indifferent behavior of the plant towards these elements (indicator) and a ratio clearly < 1 shows that the plant exclude these elements from uptake (excluder) [40].

Mean values of TF and MR indicated effective translocation of most elements in *Vitis vinifera* L. at all three sampling sites (Table 5 and Table 6). Effective translocation of Ni (Feteasca alba), Co (Feteasca alba, Feteasca regala and Italian Riesling), As (Feteasca alba, Feteasca regala and Italian Riesling), Cr (Feteasca alba, Feteasca regala and Italian Riesling) occurs from soil to grapevine roots. From roots to canes, effective translocation was recorded for Pb (Feteasca alba, Feteasca regala and Italian Riesling), Cd (Feteasca alba, Feteasca regala and Italian Riesling), Ni (Italian Riesling), Co (Feteasca regala and Italian Riesling). From canes to leaves, translocation was recorded to Cu (Feteasca alba), Pb (Feteasca alba, Feteasca regala and Italian Riesling), Ni (Feteasca alba and Feteasca regala), Co (Feteasca alba), As (Feteasca alba, Feteasca regala and Italian Riesling) and Hg (Feteasca alba, Feteasca regala and Italian Riesling), while from grapes to must, effective translocation of Cu (Feteasca alba, Feteasca regala and Italian Riesling), Zn (Feteasca alba, Feteasca regala and Italian Riesling) and Cr (Feteasca alba, Feteasca regala and Italian Riesling) was detected. For most elements, translocation coefficient between grapes-cane, must-grapes, and wine-must had values lower than 1, indicating grapevine’s specific mechanisms to block the accumulation of toxic metals in grapes [41,42,43]. The physico-chemical and biological processes that occur in the process of transformation the must into wine generates the reducing of the heavy metals concentrations, and this is demonstrated with the lower values of the analyzed metals in wine and in must as well from the values lower than 1 of the TFs [23] based on MR values, absorption of Cu, Zn, Pb from soil to roots, canes, leaves, grapes, must, and wine of all varieties of *Vitis vinifera* L. was not considerable (MR<1). In case of Cd (canes/soils), As (roots/soil and leaves/soil), Hg (canes/soil), MR value around 1 indicates that plants had an indifferent behavior against these elements. According to literature data, *Vitis vinifera* L. can be considerate as a bioaccumulator of Pb, Cu, and Zn [14,21]. Our results also demonstrated that *Vitis vinifera* L. is not a hyperaccumulator of Cu, Zn, Pb, Cd, Ni, Co, As, Cr and Hg (absorb metals above established background concentration).

### 2.6. Combining Multielement Analysis of Must and Wine for Geographical Discrimination

Elements like Mn, Cd, Li, Ba, Ca, Bi, Rb, Mg, Ag, Ni, Cr, Sr, Zn, Rb and Fe showed a high discriminatory power for geographic origin of Romanian wine, but additional new elements (Hg, Ag, As, Al, Tl, U), metal ratios (Ca/Sr and K/Rb) and ^207^Pb/^206^Pb, ^208^Pb/^206^Pb, ^204^Pb/^206^Pb, ^87^Sr/^86^Sr isotope ratios have been investigated in order to identify new tracers for geographical traceability of Romanian wines [24,26,44].

This is the first study to assess the geographic fingerprinting of wine and must samples from a polluted area (Baia Mare and Baia Sprie). The analyzed wine samples showed high concentration of elements, but not exceeding the maximum levels recommended by International Organisation of Vine and Wine (OIV 2016), except for Cu, Zn, Pb and Cd in Baia Mare and Baia Sprie. In Simleul Silvaniei, the high concentration of some elements is mostly derived from agricultural practices, fertilizers, and technological winemaking processes. Multivariate chemometric method was applied for the differentiation of must and wine intro groups based on their geographic origin. Linear discriminant analysis (LDA) was used to identify significant tracers for classification to the geographical discrimination of the wine samples.

Based on the elemental contents, cross-validation technique provided an 88.09% and 84.87% percentage of predicted membership according to the must and wine geographic origin, respectively (Supplementary Figures S1 and S3). The linear correction revealed acceptable scores for the two defined discriminant factors (F1 = 73.09%, F2 = 15.01% for must and F1 = 62.36%, F2 = 22.50% for wine). F1 mainly separates Baia Mare and Baia Sprie areas from Simleul Silvaniei and F2 separates Simleul Silvaniei from Baia Mare and Baia Sprie (Supplementary Figure S2). Among the investigated parameters, Cr, Hg, As, Cu, Zn, Pb, Ni and Cd was identified as the most significant for geographic differentiation of the must and wine from Baia Mare, Baia Sprie, and Simleul Silvaniei areas. The technique of cross-validation was applied during the set validation and the proposed model appears to be a promising chemometric approach for precise classification of wines according to their geographical origin. Thus, in both cases, the geographical regions were correctly classified with percentage between 52% and 71%.

### 2.7. Cluster Analysis

The hierarchical dendrogram for polluted sites based on elements content in sol material (Supplementary Figure S5) showed two primary clusters of the contaminated locations. The first cluster is formed of sites located in Simleul Silvaniei area, while the second one is formed of sites from Baia Mare and Baia Sprie. In terms of measure interval, the difference between the two primary clusters was significant, which suggests higher soil pollution in Baia Mare and Baia Sprie compared to Simleul Silvaniei. Both primary clusters were further divided into several new subclusters. However, the differentiation between the areas from Baia Mare and Baia Sprie was more significant than Simleul Silvaniei area. The position of an isolated subcluster which belongs to the Baia Mare area suggested that this area is the most polluted one. The dendrogram of elements in vineyard soil (Supplementary Figure S6) showed two main clusters (one isolated for As and other for the rest of elements) and numerous subclusters. The difference between primary clusters was significant, which confirmed the previous conclusion that the source of As content in soils is of geological origin, whereas the concentrations of other metals in soil are also influenced by atmospheric pollution. This was particularly obvious in the case of Cu, Pb, Zn and Cd. Similar conclusions can be formulated from analysis of the dendrogram based on element contents in grapevine roots (Supplementary Figure S7), that indicated one cluster for Ni and another cluster for the rest of elements, as well as numerous different subclusters. The dendrogram of elements in grapevine canes, leaves and grapes (Supplementary Figures S8–S10) showed two main cluster: one isolated for Hg (canes dendrogram), As (leaves dendrogram), and Cd (grapes dendrogram) and another for the rest of elements; and several different subclusters. These results also demonstrated the two possible sources of the investigated elements in these organs: soil or atmosphere. The hierarchical dendrogram for must and wine based on elements content (Supplementary Figures S11 and S12) showed two primary clusters. For must, first cluster is formed by Zn, Cu, Hg, Cd, Pb, Co and As and the second cluster is formed by Cr and Ni. For wine, first cluster is formed by Pb, Zn, Cd, Cu, Ni and the second cluster is formed by Hg, Co, Cr, As. The hierarchical dendrogram for the elements in the upper organs of grapevine (Supplementary Figure S13) also showed two main clusters: one cluster formed by Co, Ni, Hg, Cd, (grapes), Hg (canes), As (leave) and other for the rest of elements, in canes, leaves and grapes, as well as numerous different subclusters which demonstrated well a fine structure with two possible sources for the investigated elements: soil or atmosphere. The grouping of the elements confirmed that the Co, Ni, Hg, Cd, As concentrations of soil are the main source of Co, Ni, Hg, Cd, As content in the upper organs and the influence of atmospheric pollution is the highest for the group consisting of: Zn grape, Cr cane, Co leave, Cr, grape, Cr leave, As cane, that are placed furthest from the primary cluster. The combination of methods used in this study for data analysis, such as the calculation of TFs, MRs, Pearson’s correlation study, and hierarchical cluster analysis, provided a very valuable information that made feasible a multi-aspect construction of the grapevine study and can be recommended for any similar investigation.

## 3. Materials and Methods

### 3.1. Description of the Sampling Area

The present study was conducted in Baia Mare and Baia Sprie area, one of the important mining districts in Romania. The main mining activities previously developed in the area considered of nonferrous sulfidic ore extraction and processing, aiming to obtain concentrated of Pb, Cu, Zn and precious metals. After 2006, the metallurgical industry from Baia Mare and Baia Sprie has considerably diminished its activity by closing or reducing its production capacity.

Baia Mare depression is a contact depression the interposes between the Someșana Plain and the Carpathian Mountains as a lower morphological unit, from the surrounding areas, presenting a waved surface, characterized by a convergent system of valleys and interfluves. It was formed due to the tertiary tectonic movement that took to the fragmentation and sinking of the crystalline in the Northwest part of Transylvania, as well as due to the volcanic chain of the Gutin-Oaș Mountains. The metropolitan area of Baia Mare is in the NW of Romania, in a hilly region, at an altitude of 220 m above sea level, covering an area of 1250 km^2^ and having a population of more than 200.000 residents.

The Simleul Silvaniei vineyard is located in the northwest of Romania and is delimited by the Apuseni Carpathians on the south, the Someșan Plateau on the east and the Someșan Plain on the northwest, which is known geographically under the name of Silvaniei Hills. The altitude of this depression decreases from 500 m, in the accumulation area under the mountain, at 350–300 m, located in the wide part between the Măgura Șimleului and the Plopiș Mountains. Because of its position is among the northernmost vineyard in Romania. The climate of Baia Mare, Baia Sprie and Șimleul Silvaniei area falls in both moderate continental and the mountain climate categories [45].

### 3.2. Description of the soil types

According to the Romanian Soil Taxonomic Classification [46] in the investigated areas there were found: eutricambosol, typical luvosol, stagnic luvosol, gleyic luvosol, and aluviosols. Vegetation characteristic of eutricambosol soils was represented by forests partly replaced by pastures and meadows. Eutricambosols are moderate acidic with a slight difference on soil profile. Humus content is relatively high in the organic horizon (2.76–4.44%) [46]. Luvisols were represented by typical stagnic and gleyic luvosol types. They appear on a small area near metallurgical plant and are prevalent in the southern extension of the investigated areas. These soils are developed on the low plains and poorly drained terrains. Typical luvosol was present on large areas, being covered by orchards and grasslands. The Ao horizon has grey colour. The colors of the Bt horizon vary from red to brown. Soil profile was as follows: Ao-Bt-C. Stagnic and gleyic luvosol types were poor in nutrients and humus and had low natural fertility being covered by natural grasslands. Soil profile was as follows: Ao-Ea(El)-E/B-Bt-C. The Ao horizon was 15 cm thick, the brown-grey color indicating a low content of humus. The structure was granular; the texture ranging from clay loamy to clay. Aluviosols were presented only in the western proximity of metallurgical plant and were consisted of an Ao horizon of 40 cm, which on top of C horizon of alluvial deposits [46,47].

### 3.3. Sample Collection and Processing

**Figure 1:**
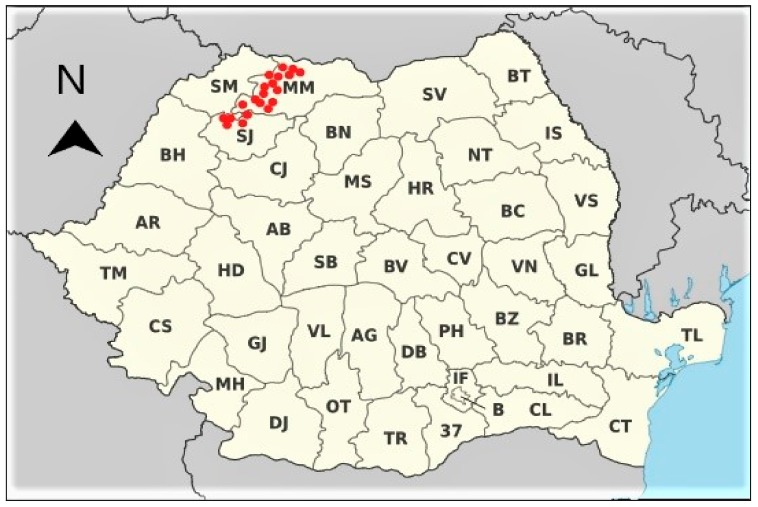
Map of the Mining and Smelting Complex Baia Mare (Northwest Romania) with the sampling points.

Soil, cane, and leave samples of grapevine were collected from Baia Mare, Baia Sprie and surrounding areas (Simleul Silvaniei) (Figure 1) during the vegetation period in May 2012. Soil samples were collected at the depth of 0–20, 20–40, 40–60 and 60–80 cm at the vineyard. Grapes of Feteasca alba, Feteasca regala, and Italian Riesling varieties were sampled one week before harvesting in August 2012. Roots (diameters <2.5 mm and >2.5 mm), canes (50–70 cane pieces of 25 cm), and leaves (50–70 fully-developed leaves from the middle part of the one-year old cane) were also collected. After removing damaged plant materials, samples were placed in sealed plastic bags and were immediately transported to the laboratory. Plant materials and soil samples were carefully processed to avoid chemical and physical interactions and analyzed by Inductively Coupled Plasma Mass Spectrometry (ICP-MS) Waltham, Massachusetts, SUA (see the Supplementary Materials).

### 3.4. Soil Sample Preparation

The soil samples (100 samples) were dried, homogenized and then passed through a 20-mesh sieve to obtain very fine particles. The method for microwave digestion using a Milestone START D Microwave Digestion System (Sorisole, Italy) was optimized in a previous work [22]: 0.25 g soil, 9 mL 65% HNO_3_, 3 mL concentrate HF and 2 mL concentrated HCl were placed in a clean Teflon digestion vessel. The vessel was closed tightly and placed in the microwave. The digestion was carried out with the program described in Supplementary Table S3. 

### 3.5. Plant Material Samples (Roots, Canes and Leaves) Preparation

The plant material samples (75 samples of roots, 113 samples of canes and 140 samples of leaves) were thoroughly washed with tap water followed by ultra-pure water using Milli-Q Integral ultrapure water-Type 1 (Darmstadt, Germany), after washing was oven-dried at 80 °C to constant weight using a FD 53 Binder (Darmstadt, Germany). The dried samples were ground using a Retsch 110 automatic mill (Darmstadt, Germany), passed through a 2 mm sieve to obtain very fine particles. The method for microwave digestion using a Milestone START D Microwave Digestion System (Sorisole, Italy) was optimized in a previous work [23]: 1 g sample of plant material, 7 mL 65% HNO_3_ and 2 mL H_2_O_2_ were placed in a clean Teflon digestion vessel. The vessel was closed tightly and placed in the microwave. The digestion was carried out with the program described in Supplementary Table S3.

### 3.6. Grape Juice Sample Preparation

Grape samples (100–110 kg/cultivar) were collected from each cultivar from 70 vines. The grapes placed in the top, middle and lower third of each vine and grapes were exposed to sun and shade [22]. In this way can achieve better homogenization of sample grapes. Feteasca regala (three samples), Feteasca alba (three samples), Italian Riesling (three samples) grape juices (must) were cold pressed manually. Before the analysis, each juice samples (50 mL) were diluted in different proportions using ultrapure water. All samples were taken in triplicates from the defined experimental plot of which had a size of 5 ha.

### 3.7. Microvinification Process

The samples of grapes were destemmed and crushed, then transferred to a microfermentor (50 L) cylindrical glass container, covered with aluminium foil to limit the effect of the light over the must) equipped with a fermentation airlock. Fermentation took place at 22–24 °C and humidity 55–60%. Afterwards wine was clarified by means of bentonite (40 g/L 1:10 dilution) and combined with SO_2_ up to 100 g/L. Then wines were allowed to cool for thirty days at −5 °C for cold stabilization [23]. Then wine samples were stored in glass bottles at 5–6 °C until the analyses. Average data from three vinifications per cultivar are reported [23].

### 3.8. Wine Sample

The wine samples were taken from freshly opened bottles and prepared by a specific organic matter digestion. 2.5 mL of wine were weighed inside Teflon digestion vessels and 2.5 mL concentrated HNO_3_ added. Teflon digestion vessels were previously cleaned in nitric solution to avoid cross-contamination. The vessels already capped were placed in a microwave oven followed by the application of the program described in Supplementary Table S3, optimized in a previous work [23]. After cooling to ambient temperature, the microwave oven was opened and the content was quantitatively transferred into a 50 mL volumetric flask and brought to the volume with ultra-pure water. All the elements were measured from these extraction solutions by ICP-MS (Waltham, Massachusetts, SUA).

### 3.9. Inductively Coupled Plasma Mass Spectrometer (ICP-MS) Analysis

Analytical measurements were performed using an inductively coupled plasma mass spectrometer (iCAP Q ICP-MS Thermo Fisher Scientific, Waltham, Massachusetts, SUA) equipped with an ASX-520 autosampler, a micro-concentric nebulizer, nickel cones and peristaltic sample delivery pump, running a quantitative analysis mode. Each sample was analyzed in duplicate and each analysis consisted of seven replicates. The gaseous argon and helium used to form the plasma in the ICP-MS was of purity 6.0 (Messer – Gases for Life, Austria). The heavy metals were measured by using a multi-element analysis after appropriate dilution using an external and standard calibration. The calibration was performed using XXICertiPUR multielement standard, and from individual standard solution of Hg. The working standards and the control samples were prepared daily from the intermediate standards that were prepared from the stock solution. The intermediate solutions stored in polyethylene bottles and glassware were cleaned by soaking in 10% *v/v* HNO_3_ for 24 h and rinsing at least ten rimes with ultrapure water (Milli-Q Integral ultrapure water-Type 1). The accuracy of the methods was evaluated by replicate analyses of fortified samples (10 µL–10 mL concentrations) and the obtained values ranged between 0.8–13.1%, depending on the element. The global recovery for each element was estimated and the obtained values were between 84.6–100.9%.

For quality control purpose, blanks and triplicates samples (*n* = 3) we analyzed during the procedure. The variation coefficient was under 5% and detection limits (ppb) were determined by the calibration curve method. Limit of detection (LoD) and Limit of quantification (LoQ) limits were calculated according to the next mathematical formulas: LoD = 3×SD/s and LoQ = 10×SD/s (SD = estimation of the standard deviation of the regression line; s = slope of the calibration curve) (Supplementary Table S4). The recovery assays for the must and wine sample of 5 µL concentration, for three replicates of this level of concentration (*n* = 3) gave the average recovery R % between 87.32% and 100.26%. The recovery for the soil and plant material samples of 5 µL concentration, for three replicates of this level of concentration (*n* = 3) gave the average recovery R % between 83.41% and 109.02%. Optimum instrumental conditions for ICP-MS measurement are summarized in Supplementary Table S3. The calibration standards were prepared from the multielement standard solution, ICP Multi Element Standard Solution XXI CertiPUR, in five concentration ranges 2.5, 5, 10, 25 and 50 µL.

### 3.10. The Determination of pH, Electrical Conductivity (EC) and Organic Matter (OM)

The pH and EC of soil samples (soil/distilled water = 1:2.5) were measured using pH meter Jenway, 3510, Keison (Chelmsford, UK) and an Electrical Conductivity (EC) meter Jenway, 3510, Keison (Chelmsford, UK), respectively. The organic matter (OM) was determined by loss-on-ignition method at 550 °C [21].

### 3.11. Reagents and Solutions

High purity ICP Multi-element Standard Solution XXI CertiPUR obtained from Merck (Darmstadt, Germany) was used for the calibration curve in the quantitative analysis. HNO_3_, concentrated HF and HCl (reagent grade from Merck, Darmstadt, Germany) and ultra-pure water (maximum resistivity of 18.2 MΏ × cm^-1^, Milli-Q Integral ultrapure water-Type 1) were used for sample preparation.

### 3.12. Statistical Analysis

Average and standard deviation were calculated, and data were interpreted with the analysis of variance (ANOVA) and the average separation was performed with the Duncan test at *p* ≤ 0.005. Pearson’s correlation coefficient was calculated using SPSS Version 24 (SPSS Inc., Chicago, IL, USA), Excel 2016 (Microsoft, New York, NY, USA) and Addinsoft version 15.5.03.3707 (Microsoft, New York, NY, USA. Value higher than 0.5 indicate a strong correlation between analyzed varieties, a positive correlation between two parameters shows that both parameters increased, and a negative correlation indicates that a parameter increased while the second one decreased and vice-versa. Linear discriminant analysis (LDA) was performed to separate the wines by region and to identify the markers with a significant discrimination value (variables with Wilk’s lambda near zero, *p* values <0.005 and higher F coefficients), using Microsoft Excel 2016 and XLSTAT Addinsoft version 15.5.03.3707. By cross-validation, we established the optimal number of parameters required to obtain a robust model.

Trace metal TF in grapevine was determined by the equation (TF_r-s_ = C_roots_/C_soils_; TF_c-r_ = C_canes_/C_roots_; TF_l-c_ = C_leaves_/C_canes_; TF_m-c_ = C_must_/C_canes_; TF_w-m_ = C_wine_/C_must_ as the ratio between roots-soil; canes-roots; leaves-canes; must-canes, and wine-must. TF > 1 indicates that grapevine translocates metals effectively from soil to plants parts [43]. The MR between the metal concentration in plant parts (C_plant_, mg/kg) and concentration in the top-soil (C_soil–m_, mg/kg) was determined according to the equation MR = C_plant_/C_soil-m_. MR > 1 indicates effective metal translocation from soil to plants parts.

## 4. Conclusions

All organs and products of *Vitis vinifera* L., except for grapes, must, and wine, provide numerous pieces of reliable information for efficient biomonitoring. Obtained data showed a very low environmental quality of the ecosystem in Baia Mare, Baia Sprie, and their surrounding areas. Furthermore, the content of most elements in plant parts is affected by airborne pollution which comes from nearby metallurgical activities, i.e., from the Cu smelter, whereas geology contributes predominately to the Ni content. Also, these results suggest that the Cu smelter is not necessarily a dominant source of pollution by As and Hg.

The most abundant elements in all plants, soil samples, must, and wine from Baia Mare and Baia Sprie areas were Cu and Zn, except for grape samples. Apparently, the investigated grapevine cultivar poses some specific means for a strong protection of grapes from high concentrations of heavy metals, while tolerates considerable amounts of heavy metals (Cu, Zn, Hg, As) in other tissues, especially in root tissue. This means that the *Vitis vinifera* L. cultivated in Baia Mare and Baia Sprie areas may have developed a wide range of cellular mechanisms that are highly effective in heavy metal detoxification and tolerance to heavy-metal-induced stress, including different tactics of restriction of metal uptake from soil as well as the retention of assimilated metals in the root tissue. Except of sporadic incidences, there were no visible symptoms of phytotoxic effects of metals, even though many of the grapevines were growing in highly polluted soils. Planting of the *Vitis vinifera* L. can be recommended in all kinds of soils that are severely polluted with heavy metals because it is a suitable candidate for phytostabilization. The plants of this climber species may also be useful as a vegetation protection barrier from considerable atmospheric pollution. At the same time, berries are safe for consumption to a large degree, which is a great advantage of this species.

## Figures and Tables

**Table 1:** The content of heavy metals in soil from areas studied (mg/kg DW) (Mean ± standard deviation) (*n* = 3).

Areas	Depth (cm)	Cu	Zn	Pb	Cd	Ni	Co	As	Cr	Hg
		M.P.L.**	M.P.L.	M.P.L.	M.P.L.	M.P.L.	M.P.L.	M.P.L.	M.P.L.	M.P.L.
Normal Values		20 mg/kg	100 mg/kg	20 mg/kg	1 mg/kg	20 mg/kg	15 mg/kg	5 mg/kg	30 mg/kg	0.1 mg/kg
Alert threshold	Susceptible	100 mg/kg	300 mg/kg	50 mg/kg	3 mg/kg	75 mg/kg	30 mg/kg	15 mg/kg	100 mg/kg	1 mg/kg
	Less Susceptible	250 mg/kg	700 mg/kg	250 mg/kg	5 mg/kg	200 mg/kg	100 mg/kg	25 mg/kg	300 mg/kg	4 mg/kg
Intervention threshold	Susceptible	200 mg/kg	600 mg/kg	100 mg/kg	5 mg/kg	150 mg/kg	50 mg/kg	25 mg/kg	300 mg/kg	2 mg/kg
	Less Susceptible	500 mg/kg	1.500 mg/kg	1.000 mg/kg	10 mg/kg	500 mg/kg	250 mg/kg	50 mg/kg	600 mg/kg	10 mg/kg
Baia Mare	0–20	2510.52 ± 164.99 e *γ*	1637.98 ± 141.78 f*γ*	3074.29 ± 201.65 d *β*	14.13 ± 1.36 d *β*	28.60 ± 3.51 a*α*	29.57 ± 1.65 a*α*	4.13 ± 0.52 abc*α*	2.25 ± 0.79 ab *αβ*	0.058 ± 0.025 a *α*
	20–40	3317.02 ± 156.30 d *β*	1317.48 ± 68.87 g*δ*	3419.25 ± 196.78 c *αβ*	13.79 ± 0.74 d *β*	27.59 ± 1.28 a*β*	19.91 ± 1.76 c *β*	3.16 ± 0.99 abcde *αβ*	1.49 ± 0.60 abcd *β*	0.053 ± 0.025 a *α*
	40–60	3146.25 ± 124.62 d *β*	2266.07 ± 93.58 e *β*	3118.06 ± 149.57 d *β*	15.66 ± 0.71 d *β*	25.43 ± 2.70 a*β*	20.21 ± 1.09 bc *β*	4.05 ± 0.45 abcd*α*	2.72 ± 0.65 a *α*	0.053 ± 0.012 a *α*
	60–80	3687.25 ± 81.82 c *α*	2734.93 ± 147.45 d *α*	3544.34 ± 166.99 bc *α*	19.78 ± 1.41 c *α*	19.52 ± 0.79 b *β*	21.98 ± 1.69 b *β*	2.49 ± 0.57 bcde *β*	2.15 ± 0.01 abc *αβ*	0.042 ± 0.024 a *α*
	Average	3165.26 ± 165.26	1989.12 ± 112.92	3288.98 ± 178.75	15.84 ± 1.36	25.29 ± 2.07	22.57 ± 1.65	3.46 ± 0.63	2.15 ± 0.51	0.052 ± 0.021
Baia Sprie	0–20	4073.87 ± 182.03 a*α*	3134.45 ± 137.89 b *βγ*	3677.95 ± 148.11 b *β*	23.25 ± 1.25 b *β*	18.00 ± 1.38 b *α*	8.41 ± 0.95 efg *β*	5.13 ± 1.22 a *α*	2.58 ± 1.25 ab *α*	0.068 ± 0.029 a *α*
	20–40	3998.09 ± 9.69 ab *αβ*	2934.62 ± 243.58 c *γ*	4262.23 ± 156.00 a*α*	19.52 ± 1.01 c *γ*	16.16 ± 1.91 b *α*	11.39 ± 1.03 d *α*	4.49 ± 2.49 ab *α*	2.35 ± 0.34 ab *α*	0.070 ± 0.017 a *α*
	40–60	3855.49 ± 58.38 bc *β*	3323.19 ± 157.94 ab *αβ*	4181.79 ± 144.30 a*α*	32.53 ± 0.99 a*α*	17.22 ± 2.33 b *α*	7.18 ± 0.99 fgh *βγ*	4.72 ± 1.01 a *α*	2.39 ± 1.15 ab *α*	0.067 ± 0.012 a *α*
	60–80	4155.95 ± 79.30 a*α*	3483.25 ± 94.11 a*α*	4127.23 ± 193.63 a*α*	32.06 ± 1.40 a*α*	18.23 ± 0.40 b *α*	6.01 ± 1.33 gh *γ*	3.49 ± 1.52 abcd *α*	1.94 ± 0.37 abcd *α*	0.052 ± 0.008 a *α*
	Average	4020.85 ± 86.85	3218.88 ± 158.88	4062.30 ± 147.01	26.84 ± 1.25	17.40 ± 1.51	8.25 ± 1.08	4.46 ± 1.56	2.31 ± 0.78	0.064 ± 0.016
Șimleul Silvaniei	0–20	621.79 ± 64.64 g*β*	76.86 ± 7.71 h *α*	12.62 ± 2.76 e *α*	0.27 ± 0.04 e *α*	7.82 ± 1.81 d *β*	5.08 ± 1.77 h *β*	2.04 ± 0.03 de *α*	0.96 ± 0.41 cd *α*	0.048 ± 0.032 a *α*
	20–40	791.71 ± 50.85 fg *α*	68.18 ± 3.09 h *α*	6.62 ± 0.45 e *β*	0.22 ± 0.15 e *α*	12.14 ± 1.53 c *α*	8.80 ± 1.05 ef *α*	2.16 ± 0.03 cde *α*	0.68 ± 0.53 cd *α*	0.060 ± 0.017 a *α*
	40–60	842.88 ± 68.11 f*α*	45.36 ± 10.19 h *β*	6.95 ± 1.68 e *β*	0.15 ± 0.06 e *α*	5.89 ± 1.17 d *β*	10.08 ± 1.24 de *α*	2.05 ± 0.57 de *α*	1.39 ± 0.21 bcd *α*	0.57 ± 0.006 a *α*
	60–80	793.69 ± 8.64 fg *α*	45.56 ± 9.79 h *β*	7.76 ± 1.83 e *β*	0.12 ± 0.10 e *α*	6.97 ± 0.49 d *β*	8.43 ± 0.93 efg *α*	1.15 ± 0.01 e *β*	1.45 ± 0.61 abcd *α*	0.034 ± 0.010 a *α*
	Average	762.52 ± 48.06	59.99 ± 6.42	8.49 ± 1.66	0.19 ± 0.09	8.20 ± 1.25	8.10 ± 1.24	1.81 ± 0.33	1.21 ± 0.44	0.050 ± 0.016
Average		2649.54 ± 88.95	1755.66 ± 93.00	2453.26 ± 109.14	14.29 ± 0.90	16.96 ± 1.61	12.97 ± 1.32	3.24 ± 0.84	1.77 ± 0.50	0.055 ± 0.066
Minimum values		621.79 ± 64.64	45.36 ± 10.19	6.62 ± 0.45	0.12 ± 0.10	6.97 ± 0.49	5.08 ± 1.77	1.15 ± 0.01	0.68 ± 0.53	0.034 ± 0.010
Maximum values		4155.95 ± 79.30	3483.25 ± 94.11	4262.23 ± 156.00	32.53 ± 0.99	28.60 ± 3.51	29.57 ± 1.65	5.13 ± 1.22	2.72 ± 0.65	0.070 ± 0.017
Sig.		***	***	***	***	***	***	**	*	in
Huzum et al., 2012 [20]		256.00	60.10	12.90	0.21	29.90	7.20	11.20	11.04 ± 0.78	–
Albulescu et al., 2009 [13]		–	–	21.90	1.77	24.55	–	–	13.32	–
Alagić et al., 2015 [21]		293.00	–	42.80	3.14 ± 0.03	16.67 ± 0.09	–	10.70 ± 0.01	–	–
Bravo et al., 2017 [22]		10.87 ± 5.10	–	16.18 ± 5.20	–	–	–	–	–	–
Bora et al., 2015 [23]		479.64 ± 53.97	69.44 ± 4.02	14.77 ± 0.74	0.45 ± 0.10	16.28 ± 2.01	9.75 ± 1.47	–	–	–
Bora et al., 2018 [24]		356.03 ± 4.36		7.00 ± 0.81	0.37 ± 0.05	5.68 ± 0.53	3.73 ± 0.48	1.29 ± 0.10	11.04 ± 0.78	0.075 ± 0.013
European Communities Council 1986		50–140	150–300	50–300	1–3	30–75	–	–	–	1–1.5
Common abundance in topsoil’s^c^		5–50	10–100	–	0.1–1	20–50	–	0.1–55	–	–
Kabata-Pendias, 2010 [25]		13–24	45–100	22–44	0.37–0.78	12.0–34	–	0–9.3	–	–
Phytotoxic levels of elements in soils ^c^		36–698	100–1.000	–	–	100	–	200	–	–

Average value ± standard deviation (*n* = 3). Greek letters are significance of difference (*p* ≤ 0.005) for the same type of soil but different profile (depth). Roman letters are significance of difference (*p* ≤ 0.05) between the depths of the soil profile. The difference between any two values, followed by at least one common letter, is insignificant. *Order of the Ministry of Waters, Forests and Environmental Protection No. 756/3 November 1997, approving the regulation on the assessment of environmental pollution, Bucharest, Romania; 1997. **M.A.L (Maximum Admissible Limit) = Normal Values. in = insignificant

**Table 2:** The content of heavy metals in plant samples (mg/kg DW)(Mean ± standard deviation) (*n* = 3).

Areas	Variety	Plant Parts	Cu	Zn	Pb	Cd	Ni	Co	As	Cr	Hg	Sig
Baia Mare	Feteasca alba	Roots	450.31 ± 13.17 d *α*	189.80 ± 1.19 c *α*	49.61 ± 6.76 g *γ*	4.27 ± 0.22 e *α*	11.66 ± 1.62 e *β*	19.56 ± 2.48 e *β*	1.11 ± 0.22 ghijk *β*	2.54 ± 0.39 c *α*	0.025 ± 0.006 defg *β*	***
		Canes	72.03 ± 2.75 g *β*	105.68 ± 5.57 ij *γ*	64.63 ± 5.17 ef *β*	4.49 ± 0.29 e *α*	8.43 ± 0.85 gh *γ*	40.31 ± 2.23 c *α*	1.33 ± 0.33 fghi *β*	0.67 ± 0.09 e *β*	0.024 ± 0.009 defg *β*	***
		Leaves	61.65 ± 1.71 g *β*	119.47 ± 1.58 hi *β*	89.64 ± 1.87 bc *α*	2.39 ± 0.02 g *β*	25.25 ± 1.14 a *α*	6.89 ± 1.01 fgh *γ*	3.13 ± 0.68 a *α*	0.40 ± 0.18 e *β γ*	0.049 ± 0.008 b *α*	***
		Grapes	8.49 ± 0.64 jk *γ*	9.60 ± 0.98 kl *δ*	6.34 ± 1.06 klm *δ*	0.69 ± 0.05 jkl *γ*	0.74 ± 0.25 o *δ*	1.26 ± 0.65 h *δ*	0.60 ± 0.30 ijkl *β*	0.13 ± 0.01 e *δ*	0.013 ± 0.002 ijk *β*	***
	Average		148.12 ± 4.57	106.14 ± 2.33	52.56 ± 3.70	2.96 ± 0.15	11.52 ± 0.97	17.01 ± 1.59	1.54 ± 0.38	0.94 ± 0.17	0.028 ± 0.006	
	Feteasca regala	Roots	460.00 ± 4.00 *α*	211.18 ± 4.81 ab *α*	60.81 ± 5.95 f *β*	7.09 ± 0.83 c *α*	12.12 ± 1.97 e *β*	32.24 ± 1.23 d *α*	0.90 ± 0.60 ghijkl *β*	3.48 ± 0.24 a *α*	0.016 ± 0.005 fghijk *γ*	***
		Canes	77.31 ± 3.76 g *β*	119.65 ± 5.75 hi *β*	68.11 ± 3.76 de *α*	5.86 ± 0.23 d *β*	6.46 ± 0.54 hijk *γ*	33.86 ± 1.92 d *α*	1.20 ± 0.44 fghi *β*	0.53 ± 0.23 e *β*	0.027 ± 0.002 def *β*	***
		Leaves	65.66 ± 1.88 g *γ*	114.52 ± 2.33 hi *β*	73.47 ± 2.64 d *α*	1.99 ± 0.02 gh *γ*	22.78 ± 0.82 bc *α*	6.17 ± 0.50 gh *β*	2.17 ± 0.50 bcd *α*	0.42 ± 0.18 e *β γ*	0.049 ± 0.009 b *α*	***
		Grapes	12.30 ± 2.39 hijk*δ*	10.13 ± 1.33 kl *γ*	6.19 ± 0.95 klm *γ*	0.58 ± 0.03 jkl *δ*	0.77 ± 0.11 o *δ*	1.08 ± 0.62 *γ*	0.36 ± 0.37 kl *β*	0.13 ± 0.03 e *γ*	0.011 ± 0.003 jk *γ*	***
	Average		153.82 ± 3.01	113.87 ± 3.56	52.15 ± 3.33	3.88 ± 0.28	10.53 ± 0.86	18.34 ± 1.07	1.16 ± 0.48	1.14 ± 0.17	0.026 ± 0.005	
	Italian Riesling	Roots	779.15 ± 4.66 a *α*	174.58 ± 3.70 d *α*	32.07 ± 1.76 i *γ*	6.42 ± 0.25 cd *α*	11.56 ± 1.40 e *β*	23.37 ± 1.40 e *α*	2.31 ± 0.76 bcd *α*	2.64 ± 1.23 bc *α*	0.011 ± 0.003 jk *β*	***
		Canes	72.93 ± 2.25 g *β*	99.45 ± 4.03 j *γ*	40.23 ± 4.24 h *β*	4.64 ± 0.13 e *β*	6.82 ± 1.28 ghij *γ*	12.42 ± 1.62 f *β*	2.09 ± 0.13 bcde *α*	0.58 ± 0.15 e *β*	0.022 ± 0.007 defghij *α*	***
		Leaves	76.82 ± 1.92 g *β*	127.58 ± 1.55 gh *β*	90.89 ± 1.55 bc *α*	2.11 ± 0.03 g *γ*	21.54 ± 0.88 c *α*	4.84 ± 0.88 gh *γ*	1.87 ± 0.80 def *α*	0.19 ± 0.11 e *β*	0.029 ± 0.006 cde *α*	***
		Grapes	9.66 ± 1.02 ijk *γ*	8.52 ± 1.25 kl *δ*	4.60 ± 0.64 klm *δ*	0.75 ± 0.15 jkl *δ*	0.60 ± 0.17 o *δ*	0.91 ± 0.06 h *δ*	0.29 ± 0.22 l *β*	0.14 ± 0.05 e *β*	0.012 ± 0.003 ijk *β*	***
	Average		234.64 ± 2.46	102.53 ± 2.63	41.95 ± 2.05	3.48 ± 0.14	10.13 ± 0.93	10.39 ± 0.98	1.64 ± 0.48	0.89 ± 0.039	0.019 ± 0.005	
Baia Sprie	Feteasca alba	Roots	661.74 ± 14.49 b *α*	160.93 ± 3.58 de *β*	84.87 ± 0.65 c *β*	3.07 ± 0.12 f *γ*	3.42 ± 0.90 lmn *β*	10.95 ± 1.26 fg *β*	1.80 ± 0.62 defg *β*	2.38 ± 0.28 cd *α*	0.020 ± 0.002 efghijk *β γ*	***
		Canes	124.56 ± 9.02 f *γ*	192.83 ± 16.12 c *α*	85.62 ± 12.97 c *β*	9.50 ± 0.83 a *α*	3.41 ± 0.38 lmn *β*	85.37 ± 7.79 a *α*	0.92 ± 0.20 hijkl *γ*	0.61 ± 0.12 e *β*	0.026 ± 0.010 def *β*	***
		Leaves	148.02 ± 16.94 e *β*	163.54 ± 26.93 d *αβ*	96.55 ± 9.07 bc *α*	6.50 ± 0.91 cd *β*	22.29 ± 1.60 bc *α*	5.31 ± 0.74 gh *β*	2.77 ± 0.18 ab *α*	0.25 ± 0.04 e *γ*	0.053 ± 0.003 b *α*	***
		Grapes	12.31 ± 1.82 hijk*δ*	8.91 ± 1.50 kl *γ*	8.91 ± 1.50 jklm *γ*	1.22 ± 0.46 ij *δ*	1.68 ± 0.69 no *β*	3.26 ± 0.69 h *β*	0.90 ± 0.07 hijkl *γ*	0.13 ± 0.02 e *γ*	0.014 ± 0.002 hijk *γ*	***
	Average		236.66 ± 10.57	131.55 ± 12.03	68.98 ± 6.05	5.07 ± 0.58	7.70 ± 0.89	26.22 ± 2.62	1.60 ± 0.27	0.84 ± 0.12	0.028 ± 0.004	
	Feteasca regala	Roots	670.51 ± 6.61 b *α*	115.61 ± 9.52 hi *β*	72.87 ± 11.00 d *β*	1.82 ± 0.44 ghi *β*	2.85 ± 0.30 mno *β*	4.78 ± 0.19 gh *β*	2.65 ± 0.96 abc *α*	2.89 ± 0.69 b *α*	0.020 ± 0.003 efghijk *γ*	***
		Canes	143.72 ± 2.46 e *β*	198.05 ± 11.47 bc *α*	93.63 ± 12.77 bc *β*	9.60 ± 0.95 a *α*	6.99 ± 0.84 ghij *β*	76.96 ± 13.65 b *α*	0.81 ± 0.17 hijkl *β*	0.47 ± 0.12 e *β*	0.029 ± 0.003 cde *β*	***
		Leaves	143.83 ± 40.38 e *β*	134.99 ± 18.42 fg *β*	95.52 ± 7.57 b *α*	9.43 ± 0.80 a *α*	24.16 ± 4.50 ab *α*	4.96 ± 1.06 gh *β*	2.50 ± 0.08 abcd *α*	0.32 ± 0.11 e *β*	0.054 ± 0.005 b *α*	***
		Grapes	13.75 ± 1.17 hijk *γ*	6.80 ± 2.47 kl *γ*	6.80 ± 2.47 klm *γ*	1.13 ± 0.53 ij *β*	2.56 ± 1.60 mno *β*	3.90 ± 1.84 h *β*	0.86 ± 0.08 hijkl *β*	0.11 ± 0.03 e *β*	0.012 ± 0.003 ijk *δ*	***
	Average		242.95 ± 12.66	113.86 ± 10.47	67.21 ± 8.45	5.50 ± 0.68	9.14 ± 1.81	22.65 ± 4.19	0.71 ± 0.32	0.95 ± 0.24	0.029 ± 0.004	
	Italian Riesling	Roots	669.15 ± 21.27 b *α*	97.66 ± 1.14 j *γ*	92.26 ± 1.11 bc *β*	1.27 ± 0.29 hij *γ*	2.31 ± 0.88 mno *γ*	6.13 ± 1.25 gh *β*	2.53 ± 0.40 abcd *α*	2.05 ± 0.56 d *α*	0.014 ± 0.002 hijk *γ*	***
		Canes	147.92 ± 21.61 e *β*	221.13 ± 6.57 a *α*	102.91 ± 0.57 a *α*	8.66 ± 0.60 b *α*	8.14 ± 1.69 ghi *β*	76.93 ± 10.85 b *α*	0.80 ± 0.20 hijkl *β*	0.55 ± 0.10 e *β*	0.025 ± 0.001 defg *β*	***
		Leaves	130.98 ± 22.64 ef *β*	147.63 ± 30.72 ef *β*	106.32 ± 14.48 a *α*	6.69 ± 0.38 c *β*	18.69 ± 1.95 d *α*	6.21 ± 1.36 gh *β*	1.92 ± 0.80 cdef *α*	0.16 ± 0.06 e *β*	0.067 ± 0.009 a *α*	***
		Grapes	14.51 ± 1.25 hijk *γ*	6.78 ± 2.14 kl *δ*	6.74 ± 1.22 klm *γ*	0.88 ± 0.10 jk *γ*	1.13 ± 0.40 no *γ*	4.60 ± 2.54 gh *β*	0.83 ± 0.17 hijkl *β*	0.12 ± 0.03 e *β*	0.013 ± 0.002 ijk *γ*	***
	Average		240.64 ± 16.69	118.32 ± 10.14	77.06 ± 4.35	4.38 ± 0.34	7.57 ± 1.23	23.47 ± 4.00	1.52 ± 0.39	0.72 ± 0.19	0.030 ± 0.004	–
Șimleul Silvaniei	Feteasca alba	Roots	10.01 ± 0.39 hijk *γ*	6.00 ± 1.49 kl *γ*	1.18 ± 0.11 m *β*	0.67 ± 0.17 jkl *β*	5.41 ± 0.74 jkl *β*	2.38 ± 0.31 h *α*	1.23 ± 0.22 fghi *β*	0.42 ± 0.24 e *α*	0.017 ± 0.005 fghijk *β*	***
		Canes	30.46 ± 2.70 h *α*	18.66 ± 0.88 k *α*	1.21 ± 0.14 m *α*	0.08 ± 0.04 kl *γ*	4.29 ± 0.92 klm *β*	3.02 ± 0.81 h *α*	0.62 ± 0.02 hijkl *γ*	0.27 ± 0.07 e *α β*	0.022 ± 0.009 defghi *β*	***
		Leaves	26.10 ± 2.79 hij *β*	12.23 ± 1.72 kl *β*	1.67 ± 0.23 m *β*	0.11 ± 0.02 kl *γ*	9.01 ± 0.39 fg *α*	1.36 ± 0.07 h *β*	2.43 ± 0.18 abcd *α*	0.10 ± 0.01 e *β*	0.038 ± 0.007 c *α*	***
		Grapes	2.31 ± 0.77 k *δ*	1.25 ± 0.53 l *δ*	0.49 ± 0.34 m *β*	1.09 ± 0.03 ij *α*	1.11 ± 0.30 no *γ*	1.44 ± 0.29 h *β*	0.17 ± 0.07 l *δ*	0.09 ± 0.02 e *β*	0.011 ± 0.002jk *β*	***
	Average		17.22 ± 1.66	9.54 ± 1.16	1.14 ± 0.21	0.49 ± 0.07	4.96 ± 0.59	2.05 ± 0.37	1.11 ± 0.12	0.22 ± 0.09	0.022 ± 0.006	–
	Feteasca regala	Roots	17.19 ± 3.42 hijk *β*	5.69 ± 0.56 kl *γ*	0.83 ± 0.60 m *β*	0.66 ± 0.44 jkl *β*	5.30 ± 1.08 jkl *β*	1.59 ± 0.29 h *β*	1.31 ± 0.14 fghi *β*	0.49 ± 0.12 e *α*	0.015 ± 0.006 ghijk *β*	***
		Canes	26.98 ± 4.05 hij *α*	20.32 ± 0.70 *α*	1.62 ± 0.06 lm *α*	0.05 ± 0.03 l *γ*	6.03 ± 0.50 ijk *β*	3.36 ± 0.33 h *α*	0.63 ± 0.05 hijkl *γ*	0.20 ± 0.06 e *β*	0.025 ± 0.004 defg *α*	***
		Leaves	31.41 ± 1.87 k *α*	16.13 ± 1.32 kl *β*	2.33 ± 0.29 lm *α*	0.13 ± 0.03 kl *γ*	10.69 ± 0.68 ef *α*	1.48 ± 0.14 h *β*	2.37 ± 0.27 bcd *α*	0.09 ± 0.04 e *β*	0.031 ± 0.007 cd *α*	***
		Grapes	3.38 ± 1.76 k *γ*	1.13 ± 0.50 l *δ*	0.40 ± 0.15 m *β*	1.30 ± 0.23 ij *α*	0.89 ± 0.12 o *γ*	1.48 ± 0.32 h *β*	0.23 ± 0.10 l *δ*	0.09 ± 0.00 e *β*	0.011 ± 0.002 k *β*	***
	Average		19.74 ± 2.78	10.82 ± 0.77	1.30 ± 0.28	0.54 ± 0.18	5.73 ± 0.60	1.98 ± 0.27	1.14 ± 0.14	0.22 ± 0.06	0.021 ± 0.005	–
	Italian Riesling	Roots	9.47 ± 0.85 ijk *β*	5.28 ± 3.58 kl *γ*	0.43 ± 0.17 m *β*	1.19 ± 0.34 ij *α*	5.58 ± 0.58 jkl *β*	1.65 ± 0.44 h *β*	1.39 ± 0.46 efgh *β*	0.51 ± 0.06 e *α*	0.013 ± 0.002 ijk *β*	***
		Canes	27.70 ± 2.10 hij *α*	20.64 ± 1.41 k *α*	1.09 ± 0.03 m *β*	0.10 ± 0.02 kl *β*	6.60 ± 1.20 hijk *β*	2.75 ± 0.78 h *α*	0.43 ± 0.02 jkl *γ*	0.25 ± 0.11 e *β*	0.023 ± 0.002 defgh *α*	***
		Leaves	22.02 ± 2.18 hijk *α*	11.77 ± 1.11 kl *β*	1.89 ± 0.14 m *α*	0.14 ± 0.01 kl *β*	8.77 ± 1.04 fgh *α*	1.43 ± 0.03 h *β*	2.25 ± 0.34 bcd *α*	0.09 ± 0.06 e *γ*	0.027 ± 0.008 def *α*	***
		Grapes	2.85 ± 0.29 k *γ*	1.11 ± 0.62 l *δ*	0.59 ± 0.28 m *β*	1.21 ± 0.67 ij *α*	1.46 ± 0.38 no *γ*	1.93 ± 0.09 h *α β*	0.13 ± 0.03 l *γ*	0.11 ± 0.02 e *γ*	0.013 ± 0.002 *β*	***
Average			15.51 ± 1.36	9.70 ± 1.68	1.00 ± 0.16	0.66 ± 0.26	5.60 ± 0.80	1.94 ± 0.34	1.05 ± 0.21	0.24 ± 0.06	0.019 ± 0.004	–
Average			148.43 ± 6.30	81.18 ± 5.05	41.27 ± 3.24	3.05 ± 0.30	8.15 ± 0.37	14.34 ± 1.81	1.37 ± 0.31	0.69 ± 0.16	0.026 ± 0.004	–
Sig.			***	***	***	***	***	***	***	***	***	–
Areas			***	***	***	***	***	***	***	***	***	–
Variety			***	***	in	***	in	***	in	*	*	–
Plant parts			***	***	***	***	***	***	***	***	***	–
Areas x Variety			***	***	***	***	***	***	*	in	**	–
Areas x Plant parts			***	***	***	***	***	***	***	***	***	–
Variety x Plant parts			***	***	***	*	***	***	***	*	in	–
Areas x Variety x Plant part			***	***	***	***	*	***	in	in	*	–
Normal range in plant tissues			4–15 ^a,b^	60 ^b^	0.1–10 ^c^	0.1–2.4 ^d^	0.05–10 ^b^	–	0.009–1.5 ^b,c^	–	–	–
				8–100 ^d^	1–13 ^d^	–	1^d^	–	–	–	–	–
Phytotoxic concentration in plant tissues			15–20 ^a,c,e^	100-500 ^b^	10–20 ^a^	5–10 ^a,c,e^	20–30 ^a^	–	>20 ^a^	–	–	–
			4–40 for leaves and 100–400 for root ^b^	150–200 ^a,c,e^	–	–	>10 ^b,e^	–	1–20 ^c^ 10–100 ^e^	–	–	–

Average value ± standard deviation (*n* = 3). Greek letters are significance of difference (*p* ≤ 0.005) for the same type of soil but different profile (depth). Roman letters are significance of difference (*p* ≤ 0.05) between the plant parts of the same variety. The difference between any two values, followed by at least one common letter, is insignificant. in = insignificant. ^a^Vamerali et al., 2010 [7]; ^b^Alloway, 2013 [31]; ^c^Kabata-Pendias, 2010 [25].

**Table 3:** The content of metal concentration in must and wine samples (Mean ± standard deviation) (*n* = 3).

Areas	Variety		Sample	Cu mg/L	Zn mg/L	Pb mg/L	Cd mg/L	Ni mg/L	Co µg/L	As µg/L	Cr µg/L	Hg µg/L	Sig
				M.P.L.	M.P.L.	M.P.L.	M.P.L.	M.P.L.	M.P.L.	M.P.L.	M.P.L.	M.P.L.	
				1 mg/L	5 mg/L	0.15 mg/L	0.01 mg/L	–	–	0.2 mg/L	–	–	
Baia Mare	Feteasca alba		Must	24.87 ± 1.77 c	12.76 ± 2.19 cd	0.36 ± 0.03 c	0.05 ± 0.01 b	1.18 ± 0.06 a	LOQ	33.06 ± 1.58 bc	634.14 ± 6.44 d	0.20 ± 0.04 abc	***
			Wine	1.47 ± 0.09 g	5.59 ± 0.12 fg	0.17 ± 0.03 cd	0.04 ± 0.03 b	0.05 ± 0.01 f	LOQ	30.40 ± 1.96 cd	652.56 ± 5.56 c	0.11 ± 0.02 e	***
	Feteasca regala		Must	20.64 ± 0.90 d	11.59 ± 2.84 cde	0.69 ± 0.05 b	0.05 ± 0.02 b	0.59 ± 0.12 b	LOQ	48.30 ± 1.27 a	642.24 ± 9.54 cd	0.18 ± 0.03 bcd	***
			Wine	1.13 ± 0.04 g	5.36 ± 0.08 fg	0.27 ± 0.02 cd	0.03 ± 0.01 b	0.07 ± 0.03 f	LOQ	37.02 ± 2.23 b	646.26 ± 4.54 cd	LOQ f	***
	Italian Riesling		Must	20.36 ± 2.81 d	14.04 ± 1.93 c	0.34 ± 0.03 cd	0.03 ± 0.02 b	0.57 ± 0.18 b	LOQ	46.35 ± 2.60 a	548.50 ± 2.37 e	0.15 ± 0.05 cde	***
			Wine	1.20 ± 0.06 g	5.80 ± 0.11 fg	0.20 ± 0.03 cd	0.02 ± 0.01 b	0.04 ± 0.02 f	LOQ	34.17 ± 1.07 bc	645.06 ± 7.58 cd	LOQ f	***
Baia Sprie	Feteasca alba		Must	32.52 ± 3.26 a	25.83 ± 3.01 a	1.14 ± 0.49 a	0.21 ± 0.11 a	0.31 ± 0.03 de	LOQ	35.68 ± 3.29 b	431.67 ± 10.03 g	0.23 ± 0.02 ab	***
			Wine	2.46 ± 1.13 g	6.04 ± 1.70 fg	0.35 ± 0.14 c	0.06 ± 0.03 b	0.08 ± 0.02 f	LOQ	26.52 ± 3.61 de	317.81 ± 11.72 i	LOQ	***
	Feteasca regala		Must	29.27 ± 2.83 b	28.50 ± 7.86 a	1.13 ± 0.14 a	0.18 ± 0.04 a	0.42 ± 0.09 cd	LOQ	50.34 ± 2.75 a	452.24 ± 23.89 f	0.25 ± 0.04 a	***
			Wine	2.12 ± 0.72 g	8.87 ± 0.52 def	0.38 ± 0.16 c	0.06 ± 0.03 b	0.06 ± 0.03 f	LOQ	21.05 ± 0.65 f	292.88 ± 5.61 j	LOQ f	***
	Italian Riesling		Must	24.08 ± 1.23 c	19.12 ± 2.32 b	1.32 ± 0.25 a	0.20 ± 0.04 b	0.41 ± 0.19 cd	LOQ	49.87 ± 2.36 a	453.56 ± 7.85 f	0.18 ± 0.06 bcd	***
			Wine	1.41 ± 0.34 g	5.98 ± 1.28 fg	0.18 ± 0.04 cd	0.03 ± 0.02 b	0.08 ± 0.04 f	LOQ	23.86 ± 2.51 ef	299.52 ± 8.77 j	LOQ f	***
Șimleul Silvaniei	Feteasca alba		Must	7.53 ± 0.06 ef	7.93 ± 0.75 ef	0.18 ± 0.05 cd	LOQ b	0.32 ± 0.05 de	LOQ	25.31 ± 3.41 ef	731.34 ± 9.84 a	0.17 ± 0.07 bcd	***
			Wine	0.25 ± 0.01 g	1.95 ± 0.06 g	LOQ d	LOQ b	0.02 ± 0.02 f	LOQ	11.59 ± 1.20 g	412.55 ± 0.61 h	LOQ f	***
	Feteasca regala		Must	8.36 ± 0.70 e	5.02 ± 0.87 fg	0.22 ± 0.03 cd	LOQ b	0.49 ± 0.04 bc	LOQ	23.58 ± 3.22 ef	711.78 ± 1.93 b	0.14 ± 0.05 cde	***
			Wine	0.12 ± 0.02 g	1.77 ± 0.07 g	0.04 ± 0.03 cd	LOQ b	0.03 ± 0.01 f	LOQ	10.38 ± 1.68 g	461.38 ± 4.37 f	LOQ f	***
	Italian Riesling		Must	5.65 ± 0.64 f	5.84 ± 0.31 fg	0.11 ± 0.03 cd	LOQ b	0.22 ± 0.03 e	LOQ	23.90 ± 3.00 ef	698.29 ± 8.59 b	0.13 ± 0.05 de	***
			Wine	0.41 ± 0.02 g	1.54 ± 0.04 g	LOQ d	LOQ b	0.02 ± 0.01 f	LOQ	13.94 ± 0.62 g	449.33 ± 6.06 f	LOQ f	***
Average			Must	19.25 ± 9.85	14.51 ± 8.41	0.61 ± 0.47	0.08 ± 0.09	0.50 ± 0.28	–	37.38 ± 11.53	589.31	0.18 ± 0.04	–
			Wine	1.17 ± 0.81	4.77 ± 2.48	0.18 ± 0.14	0.03 ± 0.02	0.05 ± 0.02	–	23.21 ± 9.77	464.15 ± 150.89	0.01 ± 0.04	–
Sig				***	***	***	***	***	–	***	***	***	–
Must		Areas		**	***	***	***	in	–	***	***	in	–
		Variety		in	in	in	in	in	–	in	in	in	–
		Areas x Variety		in	in	in	in	in	–	in	in	in	–
Wine		Areas		***	***	***	***	***	–	***	***	***	–
		Variety		in	**	*	in	in	–	in	in	***	–
		Areas x Variety		in	**	in	in	in	–	***	***	***	–
Must													
Bora et al. 2015 [23]				1.97 ± 0.78	2.70 ± 1.66	0.20 ± 0.02	LOQ	0.22 ± 0.03	–	–	–	–	–
Wine													
Bora et al. 2018 [24]				0.91 ± 0.04 mg/L	3268.00 ± 14.57 µg/L	125.35 ± 6.10 µg/L	0.39 ± 0.02 µg/L	682.82 ± 7.88 µg/L	7.77 ± 0.53 µg/L	14.26 ± 0.53 µg/L	620.04 ± 5.44 µg/L	0.58 ± 0.04 µg/L	–

Average value ± standard deviation (*n* = 3). Roman letters are significance of difference (*p* ≤ 0.05) between the plant parts of the same variety. The difference between any two values, followed by at least one common letter, is insignificant.in = insignificant. M.P.L – maximum permissible limit (OIV, 2005). LOQ for Pb: 0.0010 µg/L; LOQ for Cd: 0.0073 µg/L; LOQ for Co: 0.1215 µg/L. LOQ for Hg: 0.1379 µg/L.

**Table 4:** Pearson’s correlation between the contents of the investigated element in plants parts and distance, between the contents of elemental in plants parts and related contents in soil, and between content in individual organs.

Metal	Distance	Metal	Pearson’s correlation coefficients					
		Soil	Root	Cane	Leave	Grape	Must	Wine
Cu	–	–	–	–	–	–	–	–
Soil	−0.5148*	1.000	–	–	–	–	–	–
Root	−0.6874**	0.9942**	1.000	–	–	–	–	–
Cane	−0.6139**	0.9337**	0.8897**	1.000	–	–	–	–
Leave	−0.5106*	0.9112**	0.8616**	0.9983**	1.000	–	–	–
Grape	−0.4234*	0.9983**	0.9863**	0.9529**	0.9336**	1.000	–	–
Must	−0.6806**	0.9986**	0.9872**	0.9511**	0.9315**	0.9999**	1.000	–
Wine	−0.4786*	0.9847**	0.9603**	0.9817**	0.9817**	0.9932**	0.9925**	1.000
Zn	–	–	–	–	–	–	–	–
Soil	−0.4874*	1.000	–	–	–	–	–	–
Root	−0.6517**	0.7246**	1.000	–	–	–	–	–
Cane	−0.6542**	0.9887**	0.6133**	1.000	–	–	–	–
Leave	−0.4519*	0.9805**	0.8458**	0.9401**	1.000	–	–	–
Grape	−0.7561**	0.8136**	0.9902**	0.7175**	0.9120**	1.000	–	–
Must	−0.6325**	0.9586**	0.4985*	0.9904**	0.8841**	0.6145**	1.000	–
Wine	−0.4123*	0.9905**	0.8125**	0.9588**	0.9982**	0.8859**	0.9104**	1.000
Pb	–	–	–	–	–	–	–	–
Soil	−0.5895*	1.000	–	–	–	–	–	–
Root	−0.5587*	0.2024	1.000	–	–	–	–	–
Cane	−0.4023*	0.2490	0.9989**	1.000	–	–	–	–
Leave	−0.3655	0.4885*	0.9534**	0.9667**	1.000	–	–	–
Grape	−0.2306	0.3946	0.9797**	0.9882**	0.9945**	1.000	–	–
Must	−0.6302**	−0.4293*	0.7976**	0.7679**	0.5784*	0.6605**	1.000	–
Wine	−0.6115**	0.3369	0.9903**	0.9958**	0.9861**	0.9981**	0.7058**	1.000
Cd	–	–	–	–	–	–	–	–
Soil	−0.6003**	1.000	–	–	–	–	–	–
Root	−0.7654**	0.6589**	1.000	–	–	–	–	–
Cane	−0.5895**	0.9737**	0.8129**	1.000	–	–	–	–
Leave	−0.6012**	0.9284**	0.3321	0.8194**	1.000	–	–	–
Grape	−0.4517*	−0.3701	−0.9427**	−0.5719*	0.0017	1.000	–	–
Must	−0.3561	0.8844**	0.9338**	0.9675**	0.6476**	−0.7608**	1.000	
Wine	−0.3328	0.6310**	−0.1679	0.4377*	0.8741**	0.4873*	0.1960	1.000
Ni	–	–	–	–	–	–	–	–
Soil	0.5145*	1.000	–	–	–	–	–	–
Root	−0.5655*	0.6589**	1.000	–	–	–	–	–
Cane	−0.3624	0.9737**	0.8129**	1.000	–	–	–	–
Leave	−0.2784	0.9284**	0.3321	0.8194**	1.000	–	–	–
Grape	−0.3652	−0.3701	−0.9427**	−0.5719*	0.0017	1.000	–	–
Must	−0.6459**	0.8844**	0.9338**	0.9675**	0.6476**	−0.7608**	1.000	–
Wine	−0.7412**	0.6310**	−0.1679	0.4377*	0.8741**	0.4873*	0.1960	1.000
Co	–	–	–	–	–	–	–	–
Soil	−0.6874**	1.000	–	–	–	–	–	–
Root	−0.5166*	0.9767**	1.000	–	–	–	–	–
Cane	−0.5894*	−0.1765	0.0388	1.000	–	–	–	–
Leave	−0.4326*	0.5882**	0.7480**	0.6822**	1.000	–	–	–
Grape	−0.3621	−0.6345**	−0.4539*	0.8728**	0.2519	1.000	–	–
Must	0	0	0	0	0	0	1.000	–
Wine	0	0	0	0	0	0	0	1.000
As	–	–	–	–	–	–	–	–
Soil	−0.5632*	1.000	–	–	–	–	–	–
Root	−0.5132*	0.8541**	1.000	–	–	–	–	–
Cane	−0.4006*	0.4093*	−0.1249	1.000	–	–	–	–
Leave	−0.4539*	0.9815**	0.7387**	0.5766*	1.000	–	–	–
Grape	−0.3165	0.9527**	0.9718**	0.1126	0.8768**	1.000	–	–
Must	−0.6845**	0.9655**	0.6893**	0.6327**	0.9975**	0.8407**	1.000	–
Wine	−0.6632**	0.6529**	0.1638	0.9583**	0.7860**	0.3918	0.8276**	1.000
Cr	–	–	–	–	–	–	–	–
Soil	−0.5123*	1.000	–	–	–	–	–	–
Root	−0.5894*	0.9511**	1.000	–	–	–	–	–
Cane	−0.3360	0.9644**	0.9989**	1.000	–	–	–	–
Leave	−0.2135	0.8558**	0.9737**	0.9621**	1.000	–	–	–
Grape	−0.6884**	−0.9254**	0.7637**	−0.7924**	−0.5960*	1.000	–	–
Must	−0.7123**	−0.8724**	−0.6788**	−0.7122**	−0.4938*	0.9926**	1.000	–
Wine	−0.6054**	−0.0196	0.2901	0.2453	0.5004	0.3970	0.5058*	1.000
Hg	–	–	–	–	–	–	–	–
Soil	−0.7456**	1.000	–	–	–	–	–	–
Root	−0.7023**	0	1.000	–	–	–	–	–
Cane	−0.6648**	0.9912**	0	1.000	–	–	–	–
Leave	−0.7123**	0.9799**	0	0.9449**	1.000	–	–	–
Grape	−0.6948**	0	0	0	0	1.000	–	–
Must	−0.5123*	0.9527**	0	0.9042**	0.9942**	0	1.000	–
Wine	−0.7123**	−0.3812	0	−0.500*	−0.1890	0	−0.822**	1.000

*Correlation is significant at the 0.05 level (two-tailed); **Correlation is significant at the 0.01 level (two-tailed).

**Table 5:** The mean values of translocation factors in system soil-grape-wine.

TF*** Roots/Soils										
Variety	Cu	Zn	Pb	Cd	Ni	Co	As	Cr	Hg	–
Feteasca alba	0.41	0.20	0.05	0.53	1.00	1.30	1.02	2.68	0.94	–
Feteasca regala	0.42	0.19	0.05	0.64	0.99	2.90	1.23	3.46	0.76	–
Riesling italian	0.53	0.16	0.05	0.57	0.48	2.32	1.64	2.57	0.10	–
Average	0.45 g	0.18 h	0.05 i	0.58 f	0.82 d	2.17 b	1.30 c	2.90 a	0.60e	Cr>Co>As>Ni>Hg>Cd>Cu>Zn>Pb
STDEV*	0.07	0.02	0.00	0.06	0.30	0.81	0.31	0.48	0.44	–
RSD %**	15.25 f	12.67 g	4.39 i	9.66 h	36.23 c	37.15 b	24.01 d	16.66 e	73.01 a	Hg>Co>Ni>As>Cr>Cu>Zn>Cd>Pb
TF Canes/Roots										
Feteasca alba	0.19	0.86	1.12	1.85	0.79	0.79	0.74	0.27	1.13	–
Feteasca regala	0.20	0.99	1.21	1.70	0.93	2.98	0.56	0.16	1.57	–
Riesling italian	0.16	1.20	1.15	1.65	1.65	3.07	0.57	0.25	1.53	–
Average	0.18 i	1.02 f	1.16 d	1.73 b	1.12 e	2.28 a	0.62 g	0.23 h	1.41 c	Co>Cd>Hg>Pb>Ni>Zn>As>Cr>Cu
STDEV	0.02	0.17	0.05	0.11	0.46	1.29	0.10	0.06	0.24	–
RSD %	12.16 g	16.49 e	4.02 i	6.19 h	41.07 b	56.77 a	16.32 f	24.99 c	17.16 d	Co>Ni>Cr>Hg>Zn>As>Cu>Cd>Pb
TF Leaves/Canes										
Feteasca alba	1.06	0.94	1.24	0.64	3.81	3.81	2.73	0.50	2.00	–
Feteasca regala	0.96	0.79	1.05	0.74	3.23	0.10	2.46	0.72	1.76	–
Riesling italian	0.94	0.85	1.38	0.66	0.66	0.12	1.50	0.31	2.04	–
Average	0.98 f	0.86 g	1.22 e	0.68 h	2.57 a	1.35 d	2.23 b	0.51 i	1.93 c	Ni>As>Hg>Co>Pb>Cu>Zn>Cd>Cr
STDEV	0.06	0.08	0.17	0.05	1.67	2.13	0.65	0.20	0.15	–
RSD %	6.59 i	9.13 f	13.67 e	7.93 g	65.20 b	158. 49 a	29.10 d	40.02 c	7.76 h	Co>Ni>Cr>As>Pb>Zn>Cd>Hg>Cu
TF Grapes/Canes										
Feteasca alba	0.10	0.06	0.10	0.16	0.21	0.21	0.63	0.21	0.53	–
Feteasca regala	0.12	0.05	0.08	0.14	0.23	0.05	0.58	0.25	0.41	–
Riesling italian	0.11	0.05	0.08	0.15	0.15	0.07	0.38	0.24	0.65	–
Average	0.11 e	0.05 h	0.09 g	0.15 d	0.20 c	0.11 f	0.53 a	0.24 b	0.53 a	As>Hg>Cr>Ni>Cd>Cu>Co>Pb>Zn
STDEV	0.01	0.01	0.01	0.01	0.04	0.09	0.13	0.02	0.12	–
RSD %	6.34 i	13.21 f	14.13 e	7.89 h	20.70 d	81.45 a	24.81 b	9.25 g	22.39 c	Co>As>Hg>Ni>Pb>Zn>Cr>Cd>Cu
TF Must/Grapes										
Feteasca alba	2.78	2.18	0.10	0.11	0.57	0.00	0.05	4.52	0.02	–
Feteasca regala	1.94	2.41	0.14	0.11	0.32	0.00	0.08	4.93	0.02	–
Riesling italian	1.84	2.24	0.15	0.11	0.11	0.00	0.09	4.16	0.02	–
Average	2.19 c	2.28 b	0.13 e	0.11 f	0.34 d	0.00 i	0.07 g	4.54 a	0.02 h	Cr>Zn>Cu>Ni>Pb>Cd>As>Hg>Co
STDEV	0.51	0.12	0.03	0.00	0.23	0.00	0.02	0.39	0.00	–
RSD %	23.48 d	5.34 h	19.65 e	3.38 i	68.35 b	173.21 a	28.83 c	8.50 g	17.75 f	Co>Ni>As>Cu>Pb>Hg>Cr>Zn>Cd
TF Wine/Must										
Feteasca alba	0.07	0.30	0.33	0.38	0.09	0.09	0.79	0.85	0.23	–
Feteasca regala	0.06	0.35	0.35	0.39	0.12	0.00	0.58	0.82	0.00	–
Riesling italian	0.06	0.35	0.22	0.22	0.22	0.00	0.60	0.89	0.00	–
Average	0.06 h	0.33 c	0.30 e	0.33 d	0.14 f	0.03 i	0.66 b	0.85 a	0.08 g	Cr>As>Zn>Cd>Pb>Ni>Hg>Cu>Co
STDEV	0.00	0.03	0.07	0.10	0.07	0.05	0.11	0.03	0.13	–
RSD %	6.16 h	9.47 g	22.67 e	29.76 d	48.64 c	173.21 a	17.52 f	3.89 i	173.21 b	Co>Hg>Ni>Cd>Pb>As>Zn>Cu>Cr

STDEV* = Standard deviation; RDS %** = Relative standard deviation; TF*** = Translocation factors.

**Table 6:** The mean values of mobility ratio in system soil-grape-wine.

MR*** Roots/Soils										
Variety	Cu	Zn	Pb	Cd	Ni	Co	As	Cr	Hg	–
Feteasca alba	0.411	0.201	0.055	0.529	0.995	1.302	1.025	2.677	0.938	–
Feteasca regala	0.418	0.187	0.055	0.639	0.967	2.895	1.230	3.457	0.759	–
Riesling italian	0.534	0.156	0.051	0.566	0.477	2.317	1.637	2.571	0.105	–
Average	0.45 g	0.18 h	0.05 i	0.58 f	0.82 d	2.17 b	1.30 c	2.90 a	0.60 e	Cr>Co>As>Ni>Hg>Cd>Cu>Zn>Pb
STDEV*	0.07	0.02	0.00	0.06	0.30	0.81	0.31	0.48	0.44	–
RSD %**	15.25 f	12.67 g	4.39 i	9.66 h	36.23 c	37.15 b	24.01 d	16.66 e	73.01 a	Hg>Co>Ni>As>Cr>Cu>Zn>Cd>Pb
MR Canes/Soils										
Feteasca alba	0.076	0.174	0.062	0.981	0.782	1.023	0.758	0.725	1.062	–
Feteasca regala	0.085	0.185	0.066	1.083	0.922	8.631	0.685	0.564	1.191	–
Riesling italian	0.085	0.186	0.058	0.933	0.786	7.114	0.936	0.642	0.160	–
Average	0.082 h	0.182 g	0.062 i	0.999 b	0.830 c	5.589 a	0.793 e	0.644 f	0.805 d	Co>Cd>Ni>Hg>As>Cr>Zn>Cu>Pb
STDEV	0.005	0.007	0.004	0.077	0.080	4.026	0.129	0.080	0.562	–
RSD %	6.054 h	3.873 i	6.210 g	7.670 f	9.590 e	72.039 a	16.278 c	12.468 d	69.793 b	Hg>Co>As>Cr>Ni>Cd>Pb>Cu>Zn
MR Leaves/Soils										
Feteasca alba	0.080	0.163	0.076	0.625	2.980	3.897	2.071	0.362	2.123	–
Feteasca regala	0.081	0.145	0.069	0.802	2.978	0.896	1.685	0.407	2.099	–
Riesling italian	0.079	0.159	0.081	0.619	0.522	0.889	1.401	0.201	0.327	–
Average	0.080 h	0.156 g	0.075 i	0.682 e	2.160 a	1.894 b	1.719 c	0.323 f	1.516 d	Ni>Co>As>Hg>Cd>Cr>Zn>Cu>Pb
STDEV	0.001	0.010	0.006	0.104	1.419	1.735	0.336	0.108	1.030	–
RSD %	1.128 i	6.128 h	7.652 g	15.270 f	65.687 c	91.589 a	19.554 e	33.510 d	67.924 b	Co>Hg>Ni>Cr>As>Cd>Pb>Zn>Cu
MR Grapes/Soils										
Feteasca alba	0.008	0.011	0.006	0.159	0.165	0.215	0.480	0.153	0.568	–
Feteasca regala	0.010	0.010	0.005	0.150	0.214	0.422	0.400	0.143	0.494	–
Riesling italian	0.009	0.009	0.005	0.142	0.120	0.474	0.359	0.157	0.105	–
Average	0.009 h	0.010 g	0.005 i	0.150 f	0.166 d	0.371 c	0.413 a	0.151 e	0.389 b	As>Hg>Co>Ni>Cr>Cd>Zn>Cu>Pb
STDEV	0.001	0.001	0.001	0.008	0.047	0.137	0.062	0.007	0.249	–
RSD %	11.519 f	9.412 g	14.590e	5.588 h	28.296 c	37.006 b	14.941 d	4.860 i	63.947 a	Hg>Co>Ni>As>Pb>Cu>Zn>Cd>Cr
MR Must/Soils										
Feteasca alba	0.022	0.023	0.001	0.018	0.094	0.000	0.024	0.693	0.009	–
Feteasca regala	0.019	0.024	0.001	0.016	0.069	0.000	0.033	0.705	0.009	–
Riesling italian	0.017	0.020	0.001	0.016	0.014	0.000	0.032	0.653	0.002	–
Average	0.020 e	0.022 d	0.001 h	0.017 f	0.059 b	0.000 i	0.030 c	0.684 a	0.007 g	Cr>Ni>As>Zn>Cu>Cd>Hg>Pb>Co
STDEV	0.003	0.002	0.000	0.001	0.041	0.000	0.005	0.027	0.004	
RSD %	12.823e	9.398 g	9.761 f	7.217 h	69.963 b	173.205 a	16.990 d	3.930 i	56.428 c	Co>Ni>Hg>As>Cu>Pb>Zn>Cd>Cr
MR Wine/Soils										
Feteasca alba	0.001	0.007	0.000	0.007	0.008	0.000	0.019	0.586	0.002	–
Feteasca regala	0.001	0.008	0.000	0.006	0.008	0.000	0.019	0.578	0.000	–
Riesling italian	0.001	0.007	0.000	0.003	0.003	0.000	0.019	0.579	0.000	–
Average	0.001 e	0.007 c	0.000 f	0.006 d	0.006 d	0.000 f	0.019 b	0.581 a	0.001 e	Cr>As>Zn>Cd>Ni>Cu>Hg>Pb>Co
STDEV	0.000	0.001	0.000	0.002	0.003	0.000	0.000	0.004	0.001	–
RSD %	18.969e	11.139 f	27.186d	33.072 c	46.861 b	173.205 a	1.547 g	0.751 h	173.205 a	Co>Hg>Ni>Cd>Pb>Cu>Zn>As>Cr

STDEV* = Standard deviation; RDS %** = Relative standard deviation; MR*** = Mobility ratio.

## Supplementary Materials

The following are available online at https://www.mdpi.com/1420-3049/25/3/750/s1, The physical properties of the soils samples; Table S1. The physical properties of the soils samples (Mean ± standard deviation) (*n* = 3); Table S2. Pearson’s correlation matrix for investigated elemental in sol, plant material, must and wine; Figure S1. Correlation between analyzed parameters and the factors in discriminant analysis of must geographic origin; Figure S2. Differentiation of must according to geographic origin based on elements content; Figure S3. Correlation between analyzed parameters and the factors in discriminant analysis of wine geographic origin; Figure S4. Differentiation of wine according to geographic origin based on elements content; Figure S5. Hierarchical dendrogram for polluted sites based on element contents in soils; Figure S6. Hierarchical dendrogram for elements in vineyard soil; Figure S7. Hierarchical dendrogram for elements in grapevine roots; Figure S8. Hierarchical dendrogram for elements in grapevine canes; Figure S9. Hierarchical dendrogram for elements in grapevine leaves; Figure S10. Hierarchical dendrogram for elements in grapevine grapes; Figure S11. Hierarchical dendrogram for elements in grapevine must; Figure S12. Hierarchical dendrogram for elements in grapevine wine; Figure S13. Hierarchical dendrogram for elements in grapevine upper organs; Table S3. The program of the microwave oven Milestone START D Microwave Digestion System; Table S4. LoD, LoQ, BEC and r2 of the calibration for each element; Table S5. Instrumental (a) and data acquisition (b) parameters of ICP-MS.
