# Supplementary material for: Assessment of the Quality of Polluted Areas in Northwest Romania Based on the Content of Elements in Different Organs of Grapevine (Vitis vinifera L.)
Source: Molecules. 2020 Feb 9;25(3):750. doi: 10.3390/molecules25030750 (PMC7037287; doi:10.3390/molecules25030750)
Supplement: Supplementary file 1 [file molecules-25-00750-s001.pdf]

Table S1. The physical properties of the soils samples (Mean ± standard deviation) (n = 3)

Areas	Depth (cm)	pH	EC (µS/cm)	OM (%)
Baia Mare	0-20	7.56±0.05bβ	187.51±0.32 dβ	10.13±1.56 h δ
	20-40	7.40±0.01d γ	185.98±0.54 eγ	11.84±0.87 a α
	40-60	7.63±0.07aα	189.34±0.19 cα	10.89±0.38 e β
	60-80	7.24±0.09h δ	181.22±0.05 fδ	10.22±0.08 g γ
	Average	7.48±0.05	183.76±0.28	10.77±0.72
Baia Sprie	0-20	6.98±0.12 j γ	192.35±1.26 aα	9.32±0.84 j β
	20-40	7.12±0.03α	184.56±0.03 e δ	9.01±0.06 l δ
	40-60	7.01±0.08i β	191.00±0.23 bγ	9.24±0.18 k γ
	60-80	7.02±0.06i β	190.45±0.84 bβ	9.36±0.99 i α
	Average	7.03±0.07	189.59±0.58	9.23±0.52
Șimleul Silvaniei	0-20	7.24±0.12 hδ	123.56±0.89iγ	10.56±0.79 f δ
	20-40	7.36±0.05 eα	125.20±0.65 h β	11.25±0.34 c β
	40-60	7.29±0.06g γ	132.25±0.07 g α	11.28±0.94 b α
	60-80	7.32±0.01f β	132.51±0.04g α	11.05±0.55 d γ
	Average	7.30±0.06	128.38±0.41	11.04±0.66
Average		7.27±0.06	167.24±0.42	10.01±0.42
Minimum values		6.98±0.12	123.56±0.89	9.01±0.06
Maximum values		7.63±0.07	192.35±1.26	11.84±0.87
Sig.		**	***	***
Mihali et al., 2013[5]		6.80		
Alagić et al., 2015[6]		7.23	170.60	10.40

Greek letters are significance of difference ($p \leq 0.005$) for the same type of soil but different profile (depth). Roman letters are significance of difference ($p \leq 0.05$) between the depths of the soil profile. The difference between any two values, followed by at least one common letter, is insignificant.

Table S2. Pearson's correlation matrix for investigated elemental in sol, plant material, must, and wine

Metal	Cu	Zn	Pb	Cd	Ni	Co	As	Cr	Hg
	Pearson's correlation coefficients								
Soil									
Cu	1.000								
Zn	0.9968**	1.000							
Pb	0.9999**	0.9970**	1.000						
Cd	0.9659**	0.9834**	0.9664**	1.000					
Ni	-0.4623*	-0.4009*	-0.4749*	0.5849*	1.000				
Co	0.2508	0.1732	0.2488	-0.0084	0.4583*	1.000			
As	0.9521**	0.9733**	0.9527**	0.9988**	0.8610**	-0.0573	1.000		
Cr	0.9108**	0.8751**	0.9099**	0.7728**	0.9795**	0.6282**	0.7408**	1.000	
Hg	0.8967**	0.9290**	0.8976**	0.9807**	0.7768**	-0.2037	0.9891**	0.6338**	1.000
Root									
Cu	1.000								
Zn	0.8717**	1.000							
Pb	0.9555**	0.6883**	1.000						
Cd	0.5687*	0.8988**	0.3006	1.000					
Ni	0.0916	0.5678*	-0.2063	0.8712**	1.000				
Co	0.5652*	0.8969**	0.2966	0.9999**	0.8733**	1.000			
As	0.7098**	0.2735	0.8860**	-0.1758	-0.6365**	-0.1800	1.000		
Cr	0.9480**	0.9823**	0.8119**	0.8009**	0.4038*	0.7984**	0.4486*	1.000	
Hg	0	0	0	0	0	0	0	0	1.000
Cane									
Cu	1.000								
Zn	0.9971**	1.000							
Pb	0.9754**	0.9893**	1.000						
Cd	0.9902**	0.9979**	0.9966**	1.000					
Ni	0.2394	0.3121	0.4476*	0.3727	1.000				
Co	0.9284**	0.8976**	0.8236**	0.8673**	-0.1386	1.000			
As	0.1813	0.2551	0.3936	0.1370	0.9982**	-0.1972	1.000		
Cr	0.7284**	0.7781**	0.8615**	0.8170**	0.8396**	0.4216*	0.8058**	1.000	
Hg	0.9111**	0.8773**	0.7978**	0.8445**	-0.1821	0.9990**	-0.2402	0.3812	1.000
Leave									
Cu	1.000								
Zn	0.8862**	1.000							
Pb	0.8580**	0.9983**	1.000						
Cd	0.9952**	0.8364**	0.8035**	1.000					
Ni	0.7115**	0.9561**	0.9714**	0.6391**	1.000				
Co	0.7132**	0.9568**	0.9720**	0.6409**	0.9999**	1.000			
As	0.8821**	0.9999**	0.9988**	0.8316**	0.9586**	0.9593**	1.000		
Cr	0.4632*	0.8211**	0.8526**	0.3739	0.9523**	0.9516**	0.8260**	1.000	
Hg	0.9995**	0.8703**	0.8405**	0.9979**	0.6878**	0.6896**	0.8660**	0.4336	1.000
Grape									
Cu	1.000								
Zn	0.8587**	1.000							
Pb	0.9977**	0.8915**	1.000						
Cd	-0.4326*	-0.8335**	-0.4927*	1.000					
Ni	0.4023*	-0.1236	0.3394	0.6513**	1.000				
Co	0.6139**	0.1226	0.5590*	0.4461*	0.9697**	1.000			
As	0.9272**	0.6044**	0.8998**	-0.0636	0.7158**	0.8648**	1.000		
Cr	0.8492**	0.9998**	0.8830**	-0.8435**	-0.1418	0.1044	0.5897*	1.000	
Hg	0.9781**	0.7332**	0.9617**	-0.2353	0.5842**	0.7648**	0.9849**	0.7206**	1.000
Must									
Cu	1.000								
Zn	0.9298**	1.000							
Pb	0.8966**	0.9966**	1.000						
Cd	0.8591**	0.9871**	0.9969**	1.000					
Ni	0.2927	-0.0797	-0.1611	-0.2380	1.000				
Co	0	0	0	0	0	1.000			
As	0.9821**	0.8438**	0.7970**	0.7472**	0.4677**	0	1.000		

Metal	Cu	Zn	Pb	Cd	Ni	Co	As	Cr	Hg
					Pearson's correlation coefficients				
Cr	-0.9438**	-0.9992**	-0.9926**	-0.9800**	0.0398**	0	-0.8646**	1.000	
Hg	0.9366**	0.9998**	0.9949**	0.9840**	-0.0610**	0	0.8537**	-0.9998**	1.000
Wine									
Cu	1.000								
Zn	0.9847**	1.000							
Pb	0.9921**	0.9988**	1.000						
Cd	0.9997**	0.9887**	0.9949**	1.000					
Ni	0.9997**	0.9887**	0.9949**	0.9999**	1.000				
Co	0	0	0	0	0	1.000			
As	0.6159**	0.7438**	0.7098**	0.6355**	0.6355**	0	1.000		
Cr	-0.3130	-0.1426	-0.1914	-0.2890	-0.2890	0	0.5555**	1.000	
Hg	0.0897	0.2620	0.2139	0.1147	0.1147	0	0.8399**	0.9179**	1.000

*Correlation is significant at the 0.05 level (two-tailed); **Correlation is significant at the 0.01 level (two-tailed).

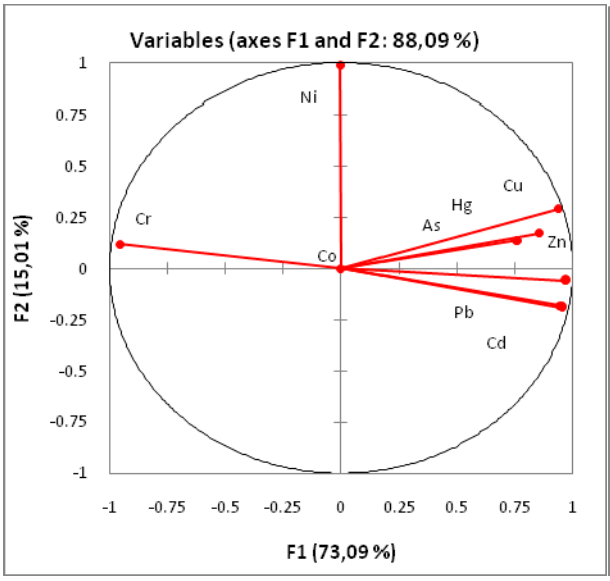

Figure S1. Correlation between analyzed parameters and the factors in discriminant analysis of must geographic origin

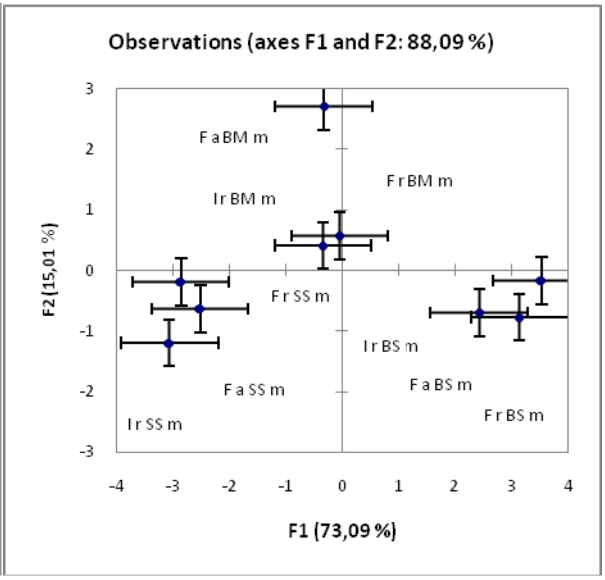

Figure S2. Differentiation of must according to geographic origin based on elements content

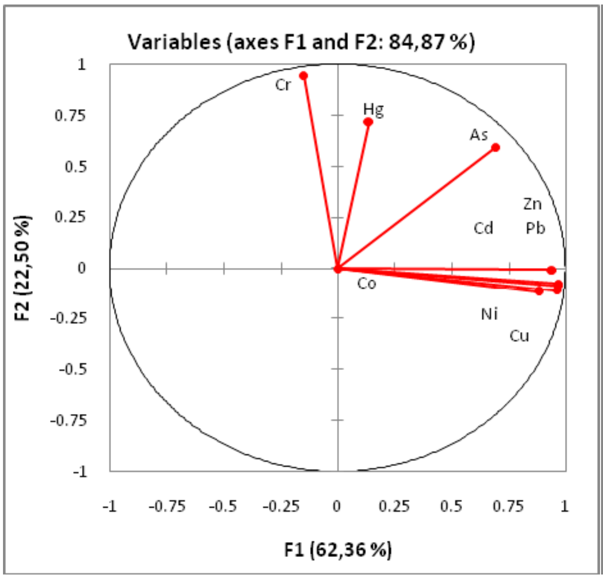

Figure S3. Correlation between analyzed parameters and the factors in discriminant analysis of wine geographic origin

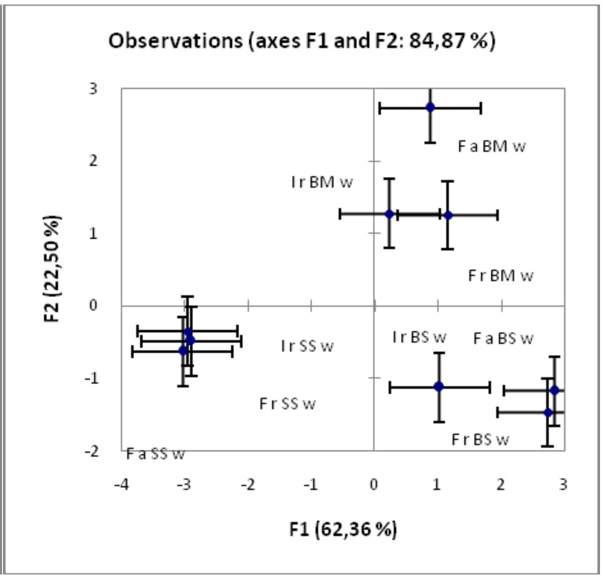

Figure S4. Differentiation of wine according to geographic origin based on elements content

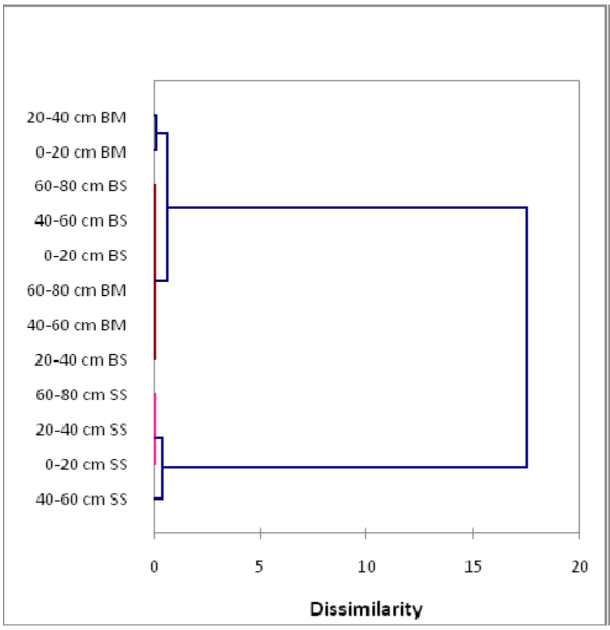

Figure S5. Hierarchical dendrogram for polluted sites based on elements content in sol

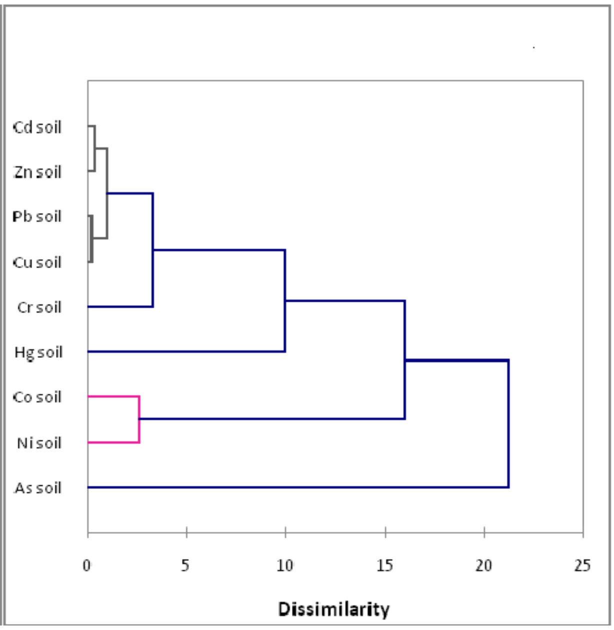

Figure S6. Hierarchical dendrogram for elements in vineyard soil

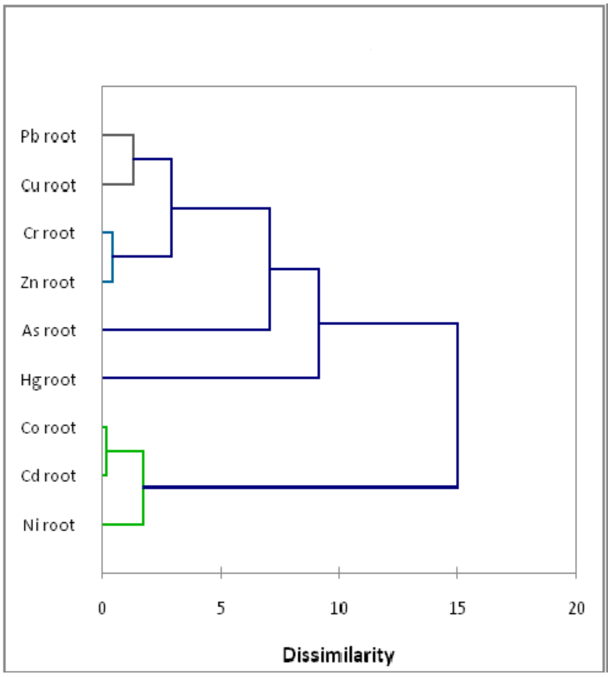

Figure S7. Hierarchical dendrogram for elements content in grapevine roots

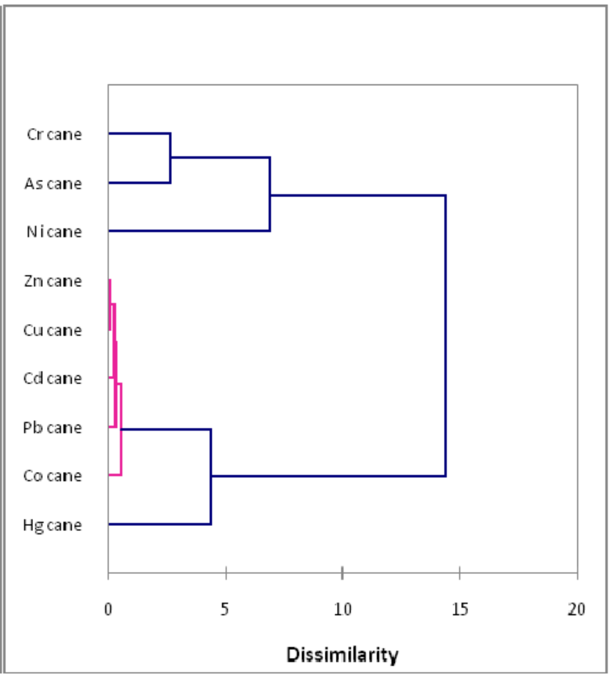

Figure S8. Hierarchical dendrogram for elements content in grapevine canes

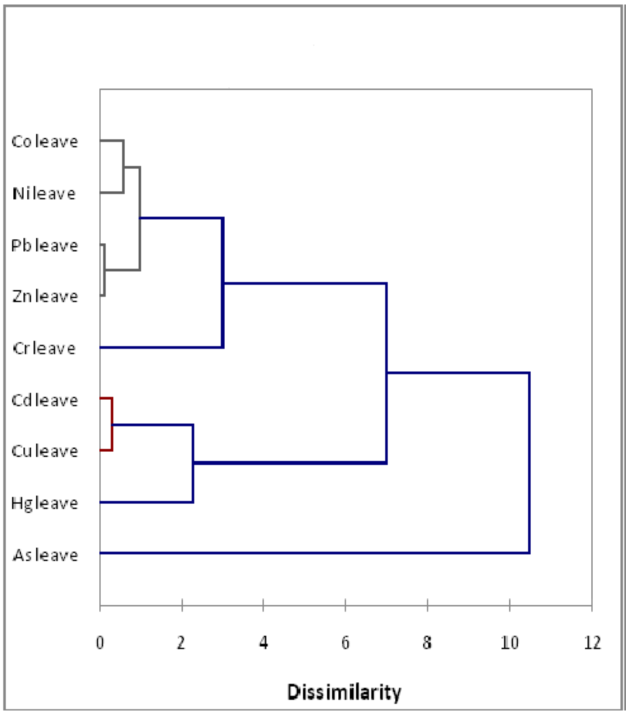

Figure S9. Hierarchical dendrogram for elements content in grapevine leaves

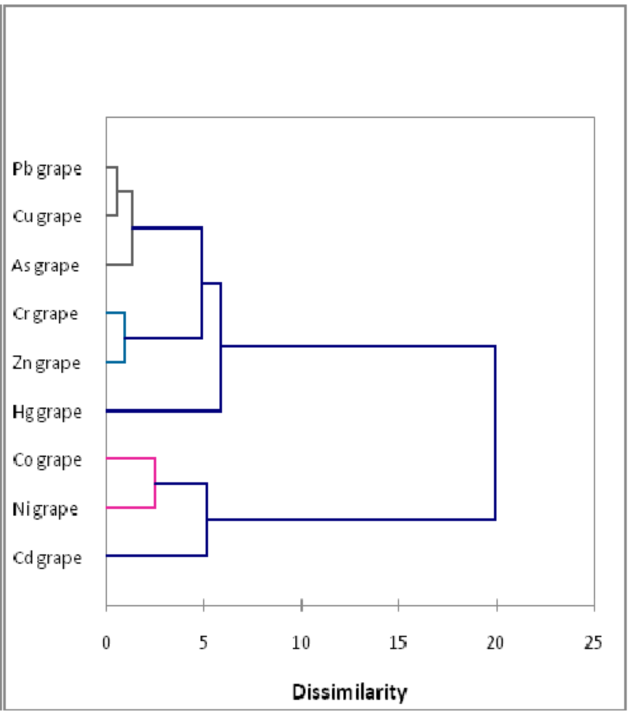

Figure S10. Hierarchical dendrogram for elements content in grapevine grapes

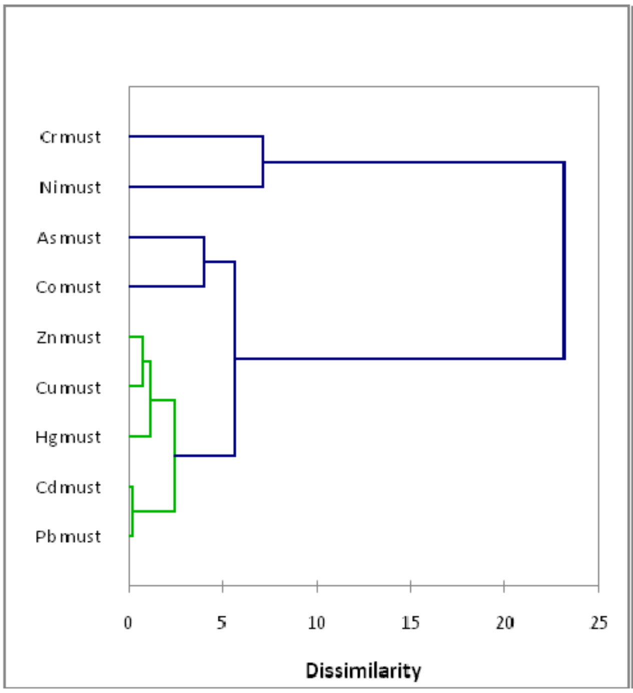

Figure S11. Hierarchical dendrogram for elements content in grapevine must

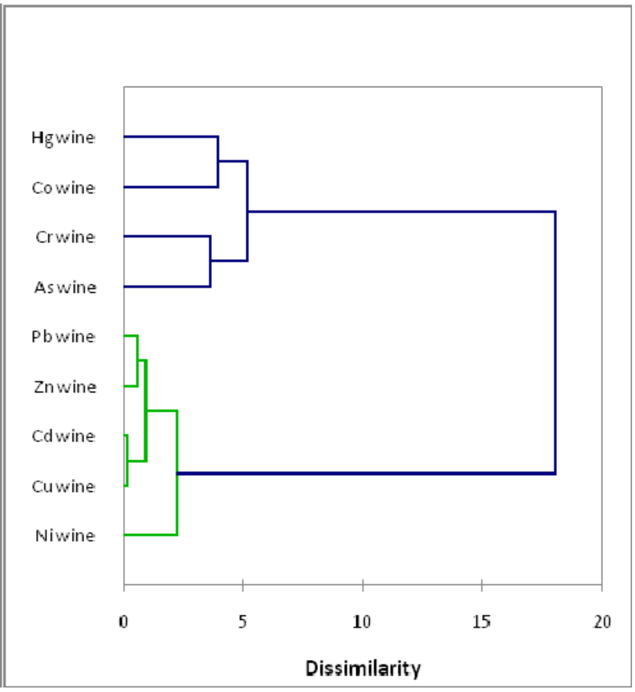

Figure S12. Hierarchical dendrogram for elements content in grapevine must

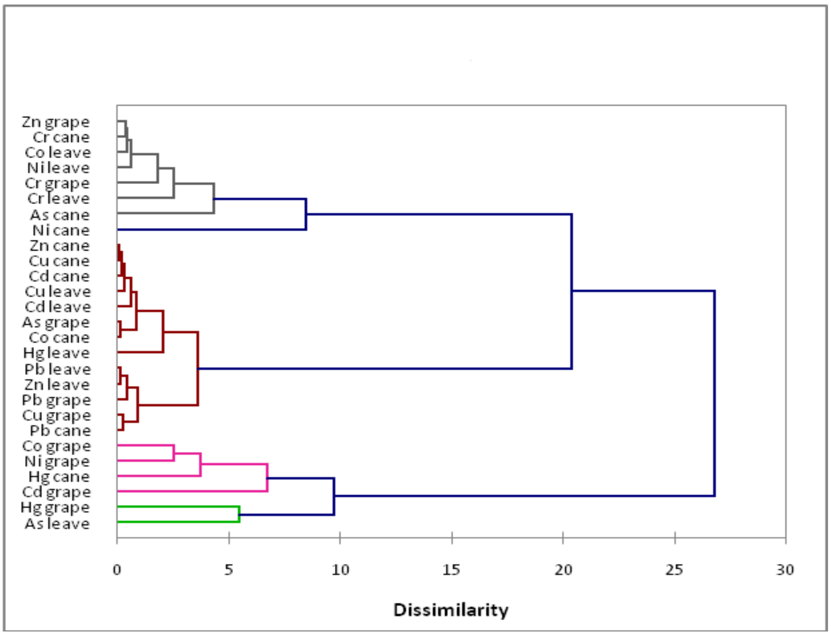

Figure S13. Hierarchical dendrogram for elements in grapevine upper organs

Table S3. The program of the microwave oven Milestone START D Microwave Digestion System

Step	Target Temp (°C)	Pressure Max (psi)	Ram Time (min.)	Hold Time (min.)	Power (%)
**Soil sample**					
1.	220	800	10	20	100
2.	30 min-cooling				
**Plant material sample (roots, canes and leaves)**					
1.	210	800	10	20	100
2.	30 min-cooling				
**Must sample**					
1.	200	800	10	20	100
2.	20 min-cooling				
**Wine sample**					
1.	200	800	10	20	100
2.	20 min-cooling				

Table S4. LoD, LoQ, BEC and r^2 of the calibration for each element

Element	r	LoD (µg/L)	LoQ (µg/L)	BEC (µg/L)
Cd	0.9999	0.0202	0.0673	0.027
Pb	0.9999	0.0003	0.0010	0.002
Hg	0.9999	0.0417	0.1379	0.128
As	0.9999	0.2335	0.7776	0.538
Co	0.9999	0.0365	0.1215	0.152
Cu	0.9999	0.0402	0.1339	0.237
Ni	0.9999	0.0591	0.1968	0.091
Cr	0.9999	1.6630	5.5378	0.636
Zn	0.9999	0.3780	1.2587	5.401

r^2 = correlation coefficient; LoD = detection limit; LoQ = Quantification limit; BEC = Background equivalent correction.

Table S5. Instrumental (a) and data acquisition (b) parameters of ICP-MS

(a) Instrumental parameters		(b) Data acquisition parameters for quantitative mode	
RF power	1.4 kW	Measuring mode	Q Cell (Collision Cell)
Argon gas flow		Point per peak	3
Nebulizer	1.0 L/min	Scans/Replicate	7
Plasma	18.0 L/min	Replicate/Sample	7
Lens voltage		Dwell time (ms)	1
Mirror lens left	37 V		
Mirror lens right	32 V		
Mirror lens bottom	31 V		
Sample uptake rate	70 s	Integration time	
